# Quantification of Förster resonance energy transfer by monitoring sensitized emission in living plant cells

**DOI:** 10.3389/fpls.2013.00413

**Published:** 2013-10-29

**Authors:** Sara M. Müller, Helena Galliardt, Jessica Schneider, B. George Barisas, Thorsten Seidel

**Affiliations:** ^1^Dynamic Cell Imaging, Faculty of Biology, Bielefeld UniversityBielefeld, Germany; ^2^Bioinformatic Resource Facility, Center for Biotechnology, Bielefeld UniversityBielefeld, Germany; ^3^Chemistry Department, Colorado State UniversityFort Collins, CO, USA

**Keywords:** Förster resonance energy transfer, fluorescence microscopy, quantitative imaging, fluorescent protein

## Abstract

Förster resonance energy transfer (FRET) describes excitation energy exchange between two adjacent molecules typically in distances ranging from 2 to 10 nm. The process depends on dipole-dipole coupling of the molecules and its probability of occurrence cannot be proven directly. Mostly, fluorescence is employed for quantification as it represents a concurring process of relaxation of the excited singlet state S_1_ so that the probability of fluorescence decreases as the probability of FRET increases. This reflects closer proximity of the molecules or an orientation of donor and acceptor transition dipoles that facilitates FRET. Monitoring sensitized emission by 3-Filter-FRET allows for fast image acquisition and is suitable for quantifying FRET in dynamic systems such as living cells. In recent years, several calibration protocols were established to overcome to previous difficulties in measuring FRET-efficiencies. Thus, we can now obtain by 3-filter FRET FRET-efficiencies that are comparable to results from sophisticated fluorescence lifetime measurements. With the discovery of fluorescent proteins and their improvement toward spectral variants and usability in plant cells, the tool box for *in vivo* FRET-analyses in plant cells was provided and FRET became applicable for the *in vivo* detection of protein-protein interactions and for monitoring conformational dynamics. The latter opened the door toward a multitude of FRET-sensors such as the widely applied Ca^2+^-sensor Cameleon. Recently, FRET-couples of two fluorescent proteins were supplemented by additional fluorescent proteins toward FRET-cascades in order to monitor more complex arrangements. Novel FRET-couples involving switchable fluorescent proteins promise to increase the utility of FRET through combination with photoactivation-based super-resolution microscopy.

## Introduction

### Background theory

**Figure 1:**
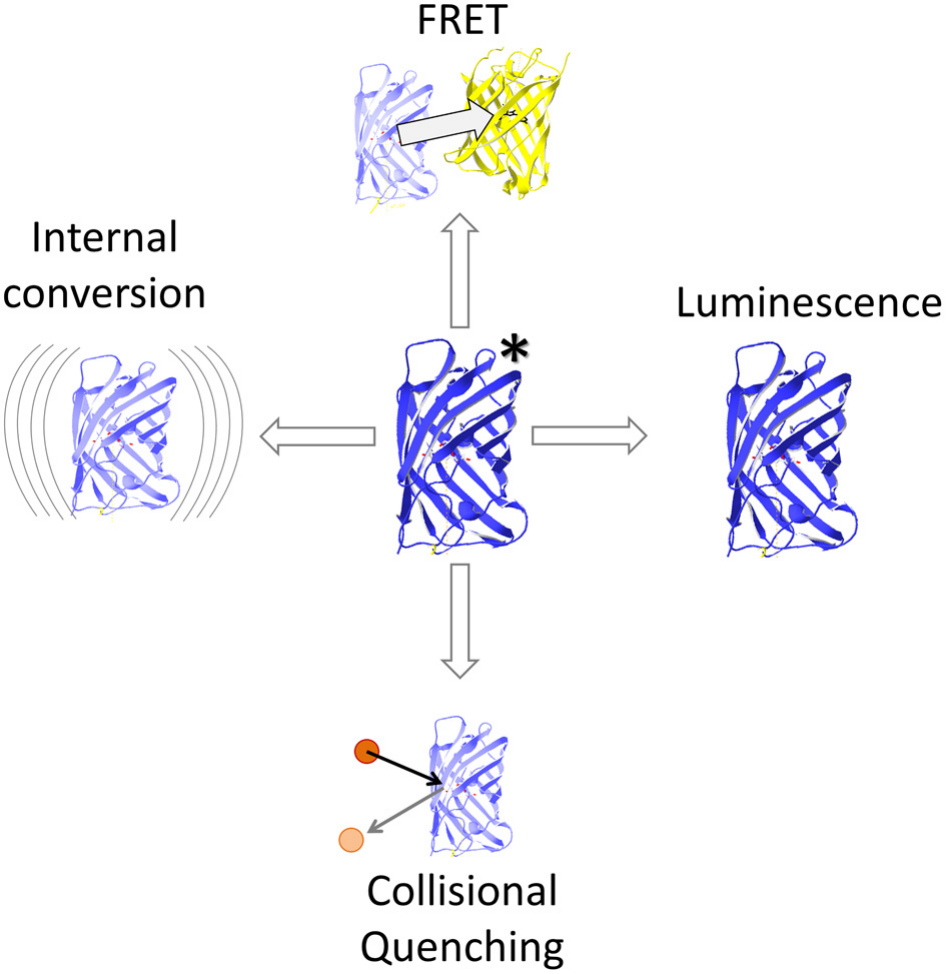
**FRET and competing events.** FRET competes with internal conversion by heat dissipation, collisional quenching e.g., with halogens and luminescence. The latter comprises fluorescence as well as forbidden transitions such as phosphorescence and delayed fluorescence.

**Figure 2:**
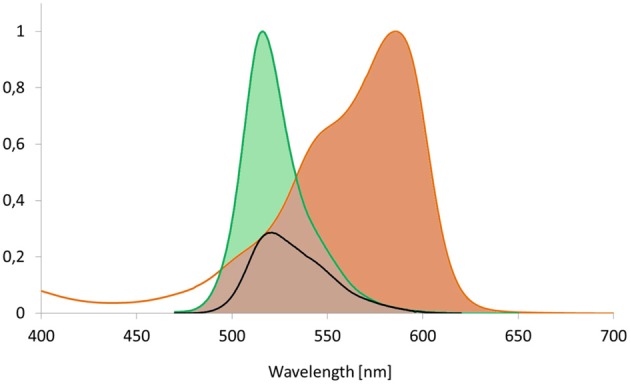
**Spectral overlap as prerequisite for FRET.** The integral of acceptor's absorption spectrum and the integral of the excited donor's emission spectrum have to show significant overlap to allow for FRET to occur. The overlap integral is shown for the fluorescent proteins Dronpa and mCherry that serve as donor and acceptor, respectively. The black line corresponds to the product of both spectra and reflects the spectral overlap.

Energy can be transferred from one molecule to another by radiationless energy transfer between two coupled dipoles. This process has been described precisely by Theodor Förster (1946, 1948) and hence has been termed Förster Resonance Energy Transfer (FRET). If the acceptor is in range of an excited donor's electric field, their dipoles can couple resulting in transfer of quantized excitation energy. More specifically, FRET describes a relaxation process from donor singlet state S_1_ to singlet state S_0_ and thus, competes with thermal relaxation (internal conversion) and with intersystem crossing toward the triplet state T_1_ followed by phosphorescence or even retrograde intersystem crossing (delayed fluorescence). The rate *k*_*T*_ of FRET contributes to the deactivation of the donor molecule (Lakowicz, 2006) and this overall deactivation rate is related to the sum of the rates of all mechanisms deactivating the excited state (Figure 1), including FRET, light emission by fluorescence, delayed light emission by phosphorescence subsequent intersystem crossing, and heat dissipation by internal conversion (Cheung, 1991; Watrob et al., 2003). The prerequisites for FRET relaxation are a close distance of the molecules, typically below 10 nm, to enable coupling of the oscillating dipole moments of both molecules in their near field, and a significant overlap of the emission spectrum of the excited molecule and the absorption spectrum of the energy accepting molecule (Figure 2), so that the donor frequency matches the acceptor frequency as the energy amounts are quantized (Table 1; Lakowicz, 2006).

**Table 1:** **Basal equations for FRET**.

(1)	**Definition of energy transfer rate *k*_*T*_**
	*R*_0_ depends on the refractive index of the medium *n*, the orientation factor κ^2^, the fluorescence quantum yield Φ_*D*_, the normalized fluorescence spectrum of the donor *F*_*D*_ (λ) and the molar absorptivity of the acceptor ε_*A*_ (λ), and the wavelength λ in cm: $R_{0}=\frac{9000\text{ ln}10\;\kappa^{2}\Phi_{D}}{128\pi^{5}n^{4}N_{A}}\int_{0}^{\infty }\frac{F_{D}\left(\lambda \right)\varepsilon_{A}\left(\lambda \right)d\lambda }{\lambda^{4}}$
(2)	**Distance-dependency of energy transfer efficiency *E***
	The efficiency *E* of energy transfer is the product of *k*_*T*_ times the unperturbed donor lifetime τ_*D*_ and varies as the inverse sixth power of the ratio of the distance *R* between donor and acceptor and the Förster radius *R*_0_: $E=\frac{k_{T}\tau_{D}}{1+k_{T}\tau_{D}}=\frac{1}{1+{\left(R/R_{0}\right)}^{6}}$
(3)	**Definition of energy transfer rate *k*_*T*_**
	*k*_*T*_ depends on the Förster radius *R*_0_, the distance *R* separating the chromophores and the unperturbed donor fluorescence lifetime τ_*D*_: $k_{T}=\frac{1}{\tau_{D}}{\left(\frac{R_{0}}{R}\right)}^{6}$

FRET also requires that the absorbing molecule undergoes a singlet-singlet transition. The efficiency *E* of energy transfer is related to the sixth power of the ratio of the distance *R* between donor and acceptor and the Förster radius *R*_0_ (Table 1). The Förster radius *R*_0_ corresponds in turn to the critical distance between two fluorophores at which the energy transfer is half-maximal (Hink et al., 2002). *R*_0_ is usually in the range of 1.5–6 nm and depends on factors including quantum yield of the donor, absorption of the acceptor and spectral overlap integral and on an orientation factor κ^2^ (Table 1; Patterson et al., 2000; Lakowicz, 2006; Lam et al., 2012).

**Figure 3:**
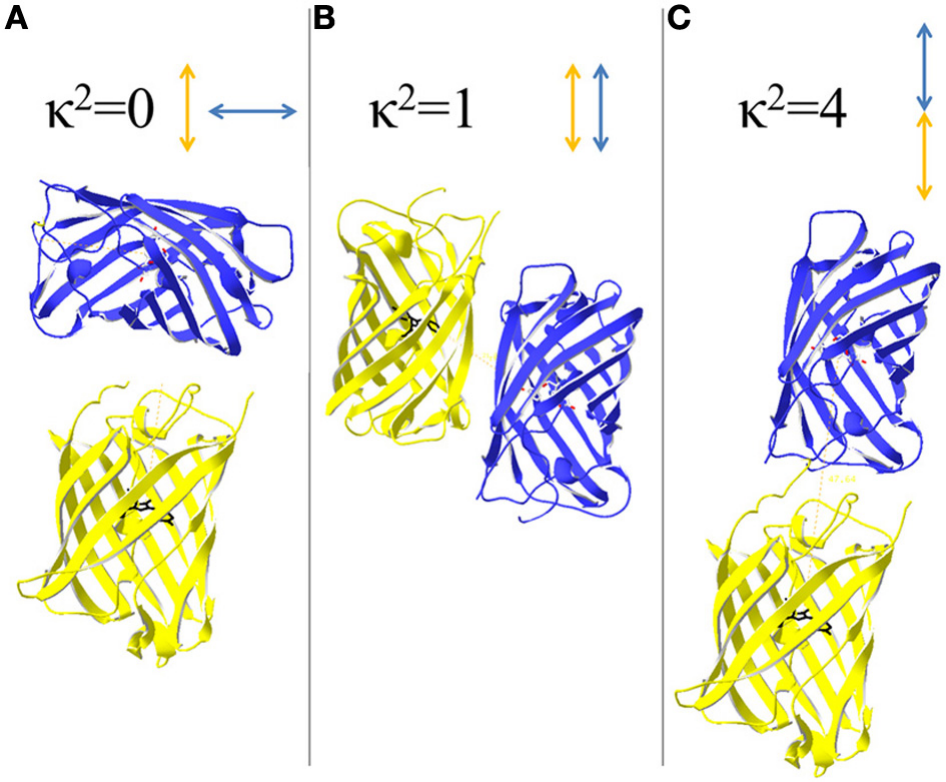
**Orientation of donor and acceptor and its influence on the orientation factor κ 2.** κ^2^ depends on the relative arrangements of excited donor's electric field and acceptors absorption dipole. **(A)** A perpendicular arrangements of the transition dipoles results in κ^2^ = 0 and prevents energy transfer between donor and acceptor. **(B)** If the dipoles are arranged side-by-side, κ^2^ becomes 1. **(C)** A head to tail arrangement of the dipoles favors FRET as κ^2^ = 4, the highest value that is possible for κ^2^.

The influence of κ^2^ becomes significant if rotational relaxation is slower than the fluorescence lifetime of the donor. κ^2^ varies in a range of 0–4 being 0, if the electric field of the excited donor and acceptor's absorption dipole are perpendicular, and 4, if they are parallel and head to tail orientated (Figure 3). The probability of possible arrangements favors a κ^2^ = 0 and there is only low probability for κ^2^ = 4 (Vogel et al., 2012). For the calculation of *R*_0_ it is assumed that rotational diffusion of the dyes is faster than the donor's fluorescence lifetime so that κ^2^ = 2/3. To this end, it is a helpful requirement if the donor is a rather small molecule allowing for fast rotation and donor and acceptor are not linked to each other so that the orientation is not fixed. For fluorescent proteins the rotation correlation time is about 20–30 ns whereas the fluorescent lifetime is in a range of 1–3 ns (Vogel et al., 2012). Thus, the assumption that κ^2^ = 2/3 appears not applicable for the calculation of *R*_0_ of fluorescent protein FRET-couples, but actually no alternative is available. Thus, the calculated *R*_0_-values are useful for comparison of FRET-pairs, if it is kept in mind that calculated distances do not correspond to the real situation. Usually, *R*_0_ is determined based on Equation 1 (Patterson et al., 2000). Calculations based on the acceptor's excitation spectrum instead of its absorption spectrum can also be performed (Rizzo et al., 2006), although this ignores possible dark states of the acceptor. For fluorescent protein couples *R*_0_ can also be determined by examining fusion constructs of donor and acceptor possessing a linker identical to that of an ECFP/EYFP fusion protein of known *R*_0_ (He et al., 2005). Thus, new *R*′_0_-values can be back-calculated from the known ECFP-EYFP distance *R*_0_ and the measured FRET-efficiency for the couples:

(4) $$R_{0}^{\prime }=\frac{R}{\sqrt[{6}]{\frac{1}{E}-1}}$$

**Figure 4:**
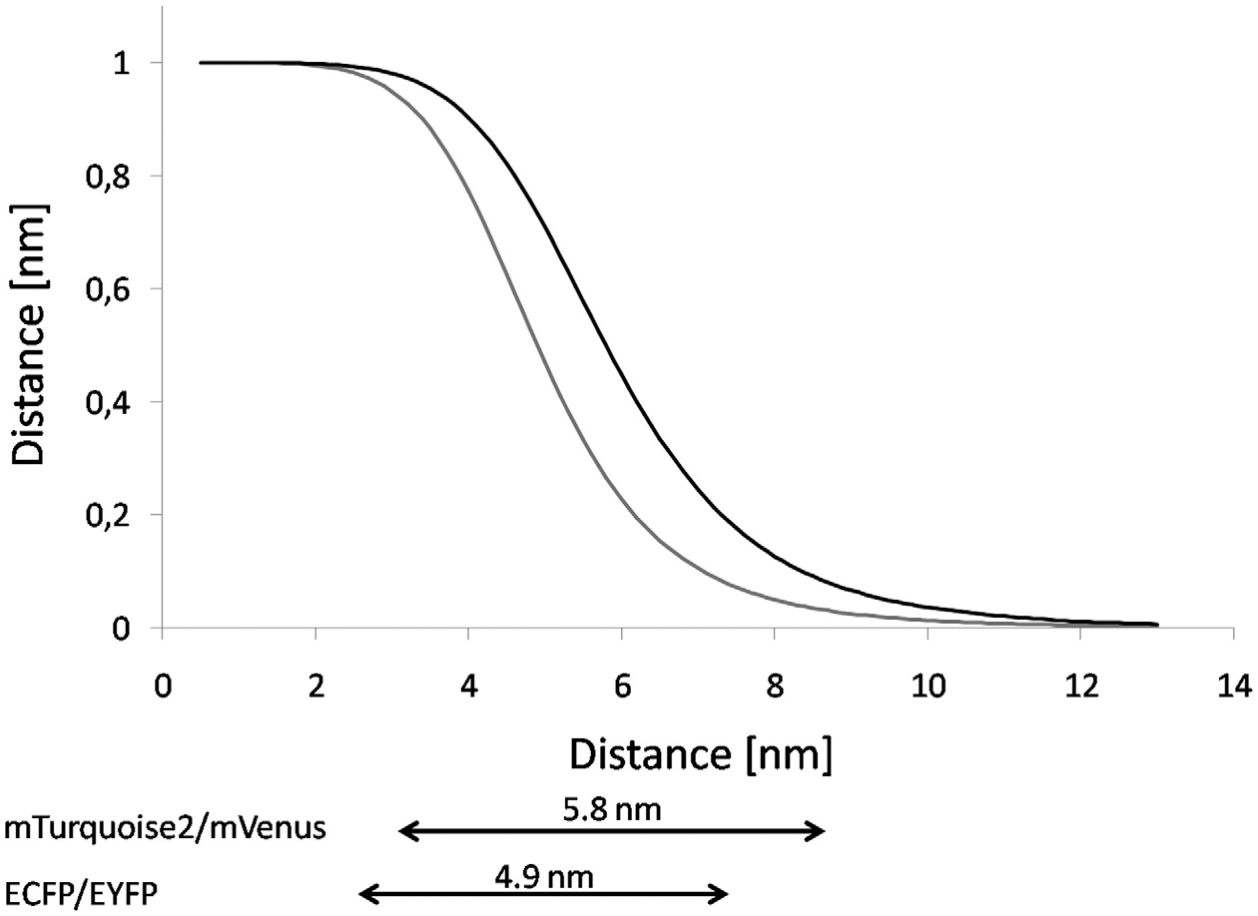
**Comparison of Distance-dependency of ECFP/EYFP and mTurquoise2/mVenus.** Based on the *R*_0_-values the FRET-efficiency was plotted against the distance. The graph shows the curves for the FRET-pairs ECFP/EYFP (gray line, source of *R*_0_: Patterson et al., 2000) and mTurquoise2/mVenus (black line, source of *R*_0_: Goedhart et al., 2012). Underneath, the dynamic range is given for both FRET-pairs. The dynamic range corresponds to 0.5 *R*_0_ − 1.5*R*_0_.

The distance range that is accessible through FRET-measurements is ~0.5 *R*_0_ ≤ *R* ≤ 1.5 *R*_0_ (Gadella et al., 1999) (Figure 4). If *R* is two times of *R*_0_, the FRET-efficiency becomes less than 0.016 and thus negligible, if *R* = 0.5 *R*_0_, the FRET efficiency becomes larger than 0.984 (Vogel et al., 2012). The higher the spectral overlap and wavelength range, the higher is the Förster radius of a given FRET-pair (Patterson et al., 2000). Also a high quantum yield of the donor yields increased *R*_0_ (Goedhart et al., 2007; Lam et al., 2012). Furthermore, *R*_0_ is sensitive to acceptor stability since blinking of the acceptor affects *R*_0_ (Vogel et al., 2012). In the case of multiple (n) acceptors proximal to a single donor, the operational *R*_0_ becomes n-times *R*_0_ (Jares-Erijman and Jovin, 2003).

The rate of FRET can be estimated both from the loss of fluorescence of the donor or an increase of fluorescence of an acceptor molecule. Alternatively, FRET decreases the lifetime of donor's excited state τ_*D*_ and results in a decrease of polarization of the emitted light (Lidke et al., 2003; Lakowicz, 2006). In the life sciences a misleading differentiation between FRET and bioluminescence resonance energy transfer (BRET) has arisen, although both represent FRET (Gandía et al., 2008). Therefore, RET was suggested to be used for FRET as the underlying phenomenon, FRET if the donor is a fluorophore, and BRET if bioluminescence is involved (Lakowicz, 2006).

The most important feature of RET for analysis of protein-protein interactions is the distance dependency. RET occurs in the range of ~0.5–10 nm (Clegg, 2009) and the diameter of a globular protein with a molecular weight of 30 kDa is ~3 nm so that the distance range critical for RET matches the dimension of proteins and turns RET to be a suitable tool for the analyses of conformational dynamics and interactions of proteins (Hink et al., 2002).

## Fluorescent proteins for FRET

**Figure 5:**
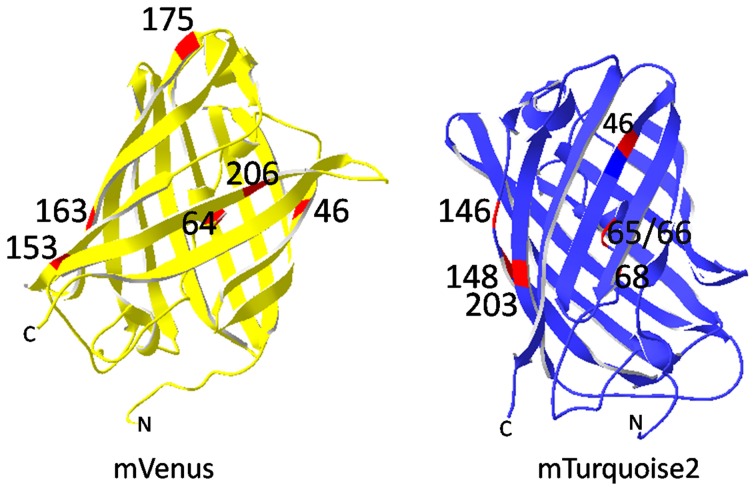
**Mutations resulting in mVenus and mTurquoise2.** mVenus bears mainly folding mutations (F64L, M153T, V163A, S175G) and F46L for optimized oxidation of the fluorophore. A206K turned Venus monomeric. Cyan emission of the mVenus derivative mTurquoise was achieved by the mutations L46F, Y203T, T65S, Y66W, and N146I. The mutations are highlighted in red. Images were processed with SwissPdb Viewer (Guex and Peitsch, 1997).

The discovery of various fluorescent proteins and the engineering of spectrally distinct variants and their improvement regarding photostability, folding efficiency, codon usage, quantum yield, insensitivity to the cellular environment and monomeric forms has enabled non-invasive FRET-measurements in living plant cells. In particular in plants, the employment of the green fluorescent protein was delayed in comparison to its use in mammals due to cryptic splicing resulting in a non-functional protein (Haseloff et al., 1997). The application of fusions of fluorescent proteins in living cells is still challenging due to differences in the sensitivity of fluorescent proteins to the (sub-)cellular environment, sensitivity of detectors that demands high expression levels, expression of proteins in cell types that do not provide their native environment, and required tolerance of proteins to N- or C-terminal fusions (Duncan, 2006). The first described FRET-pair consisted of GFP and its blue-shifted variant blue fluorescent protein (BFP) (Cubitt et al., 1995). This FRET-pair suffered from the low photostability and quantum yield of BFP (Miyawaki and Tsien, 2000), so that the combination of cyan fluorescent protein (CFP) and yellow fluorescent protein (YFP) appeared more promising. However, CFP as well as YFP were found to have major limitations in their applicability due to YFP's sensitivity to pH and halides affecting YFP's absorption, and CFP's multiple fluorescent states and its pH-dependent and low quantum yield (Miyawaki and Tsien, 2000). The pH-sensitivity of YFPs is tightly coupled to halides binding (Seward et al., 2013). In contrast to BFP-GFP this FRET-pair has been subjected to re-engineering in the recent years resulting in vastly improved variants. Basically, the folding mutations F64L, V68L, S72A, M153T, V163A, and S175G resulted in faster maturation especially at 37°C, reflecting folding mutations aimed at enhanced maturation in mammals. The extent to which these mutations are of relevance for an expression in plant cells is unclear. EYFP has been improved to yield halide- and pH-insensitive monomeric variants such as Citrine that bears the additional mutation Q69M conferring reduced sensitivity to acidosis and halides (Heikal et al., 2000; Griesbeck et al., 2001). Venus was designed for fast and complete folding so that it mainly contains the folding mutations F64L, M153T, V163A, S175G but also the mutation F46L resulting in accelerated oxidation of the fluorophore again at 37°C (Nagai et al., 2002). Recently, the kinetics of halide binding have been analyzed and confirmed the reduced halide affinity of Citrine and Venus in comparison to YFP (Seward et al., 2013). Finally, the mutation A206K affects the dimerization and turns Venus monomeric (mVenus, Figure 5). In SYFP2 the mutation V68L was reversed in the background of mVenus and resulted in a slightly brighter fluorescent protein (Kremers et al., 2006). In the case of ECFP, donor fluorescence lifetime measurements revealed a bi-exponential decay curve that hampers data evaluation, so that following improvements gained at a mono-exponential decay. Further aims were increasing the quantum yields and the absorption coefficients of CFPs. Two branches of CFPs were designed that involve similar mutations but that based on different GFP-derivatives: On the one hand CFPs of the Cerulean-branch are based on ECFP and on the other hand, CFPs derive from the SCFP-branch. The latter rely on mVenus (Kremers et al., 2006). The variant Cerulean is characterized by the mutations S72A, Y145A, and H148D and shows a significant increase in brightness compared to ECFP. Also the quantum yield and the absorption coefficient were improved. Both these aromatic amino acid residues were responsible for two different conformational states that caused the bimodal behavior of ECFP. Consequently, their replacement resulted in a CFP that showed mono-exponential decay (Rizzo et al., 2004). Nevertheless, Cerulean undergoes reversible photo-switching that strongly affects long-term measurements and bleaching experiments. Next improvements relied on mutations in β-strands 7 and 8 (S147H, D148G, K166G, I167L, R168N, H169C) and reversing the early mutation S65T to the wildtype serine that lead to Cerulean3 (Markwardt et al., 2011). Cerulean3 shows high photostability and reduced photoswitching behavior, a high quantum yield of 0.87, and only a slightly reduced absorption coefficient compared to Cerulean. Reversing only S65T stabilized the H-bonding status of the hydroxyl group and resulted in a Cerulean with increased photostability and pH-resistance, high quantum yield, and reduced reversible photoswitching (Fredj et al., 2012).

The second branch of CFPs originates from the YFP mVenus. The conversion of mVenus into SCFP1 required reversing the mutations F46L and T203Y and introduction of G65T, Y66W, and N146I. SCFP1 is characterized by a slightly red-shifted emission, low quantum yield, and short fluorescence lifetime. Next, the mutation V68L was reversed so that the red-shift of the emission disappeared and the quantum yield was improved by introducing H148D as described for mCerulean before. The resulting variant SCFP3A has an improved quantum yield of 0.56 and a slightly elongated lifetime (Kremers et al., 2006). Again, mutation S65T was reversed to increase the quantum yield to 0.84 in mTurquoise (Goedhart et al., 2010). Based on the crystal structures of SCFP3A and mTurquoise the amino acid residue Ile146 was identified as target for further improvement. Replacing it by phenylalanine creates a network of van der Waals forces stabilizing the fluorophore. The novel mTurquoise2 (Figure 5) has a unique quantum yield of 0.93, a fluorescence lifetime of 3.8 ns, is highly photostable in living cells and matures faster than mTurquoise (Goedhart et al., 2012).

An alternative CFP is the monomeric teal fluorescent protein (mTFP1) from *Clavularia* that shows a quantum yield and photostability superior to Cerulean even in its original version cFP484. Further improvements resulted in the present monomeric form mTFP1 with an absorption coefficient of 64,000 Mol^−1^cm^−1^ and a quantum yield of 0.85 (Ai et al., 2006). Taken together with its absorption maximum at 462 nm that fits quite well with 458 nm laser lines, mTFP1 appears to be a promising but up to now only rarely applied CFP of high potential for FRET applications.

Pairs of green and red fluorescent proteins represent another group couples commonly applied in FRET-analysis. These provide the advantage of a high *R*_0_ due to the increased wavelength of the spectral overlap, so that the *R*_0_ ranges up to 6.4 nm for the FRET-pair mKo/mCherry (Goedhart et al., 2007). Initially, GFP and DsRed were employed but DsRed proved to be unsatisfactory in FRET-analyses due to its slow maturation (in the range of days) accompanied by a yellowish intermediate, a complex absorption spectrum and a strong tendency to oligomerization, although the intermediate state can be counteracted by pulsed expression followed by an elongated incubation (Baird et al., 2000; Mizuno et al., 2001; Erickson et al., 2003). Intermediate state and oligomerization combine to yield intermolecular FRET potentially interfering with FRET-measurements using DsRed as acceptor, also dark states were observed for DsRed that affect FRET (Blum et al., 2011). Nevertheless, many improvements have been accomplished for DsRed and have resulted in a complete family of spectrally distinct fluorescent proteins, the mFruits-family (Shaner et al., 2004). Initially, mutations addressed the folding efficiency and aimed at monomeric forms of DsRed. The final product with altogether 33 mutations was the monomeric red fluorescent protein 1 (mRFP1) that folds 10 times faster than DsRed (Campbell et al., 2002). Unfortunately, the improvements were achieved on the expense of the quantum yield. Whereas DsRed has a high quantum yield of 0.79 it dropped to 0.25 in mRFP1. Addition of the N- and C-termini of GFP improved its tolerance to N- and C-terminal protein fusions and mutations in the environment of the fluorophore resulted in novel red fluorescent proteins such as dTomato, mStrawberry, and mCherry, but also in the yellow to orange fluorescent proteins mBanana and mOrange (Shaner et al., 2004). In the recent years mCherry became frequently applied although it has the disadvantage of a very low quantum yield of only 0.22 and two fluorescent states were reported for mCherry that result in a biexponential fluorescence decay with EGFP as donor (Shaner et al., 2004; Wu et al., 2009). But its maturation half time of 15 min is superior to any other fluorescent protein and mCherry showed extremely high photostability in single molecule analysis (Shaner et al., 2004; Seefeldt et al., 2008). Another red fluorescent protein that served as FRET-acceptor is mRuby2, an improved monomeric variant of eqFP611 with a comparatively high quantum yield of 0.38 and an absorption coefficient of 113,000 Mol^−1^cm^−1^ (Lam et al., 2012).

Mostly, EGFP serves as donor for red fluorescent proteins (Erickson et al., 2003; Peter et al., 2005; Padilla-Parra et al., 2008, 2009) due to its monoexponential fluorescence lifetime decay and insensitivity to photobleaching, high quantum yield and short maturation time (Padilla-Parra et al., 2009). The blue-shifted excitation spectrum of the neutral phenol GFP-variant T-Sapphire shows negligible acceptor spectral bleed through (ASBT) in combination with orange and red fluorescent proteins (Mizuno et al., 2001; Zapata-Hommer and Griesbeck, 2003; Bayle et al., 2008), but blue light excitation likely results in high autofluorescence background in plant cells.

**Figure 6:**
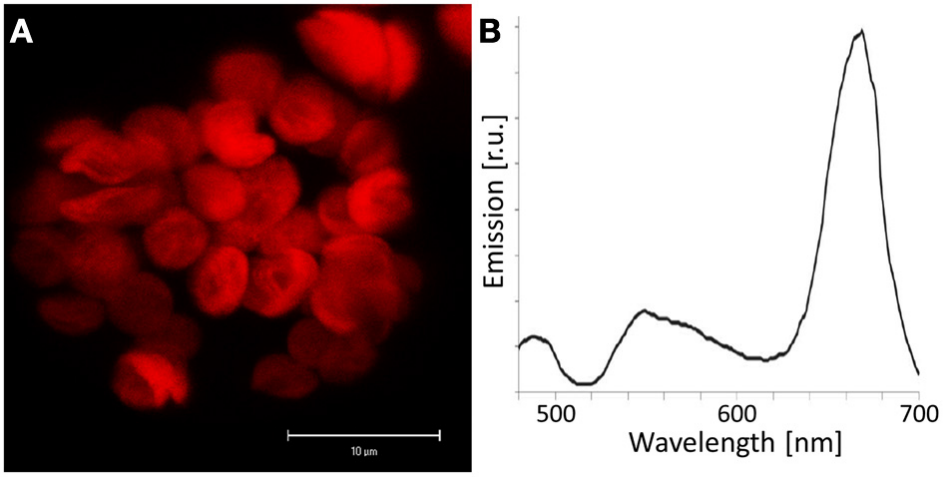
**Chlorophyll autofluorescence in Arabidopsis protoplasts. (A)** Autofluorescence was detected in the range of 650–700 nm using a 458 nm laser line for excitation by confocal laser scanning microscopy of an Arabidopsis mesophyll protoplast. A z-stack was performed and an average projection is shown. The detected emission is restricted to chloroplasts. **(B)** Subsequently, emission spectra of mesophyll protoplasts were recorded by CLSM. The minimum of emission at 514 nm was caused by the applied main beam splitter that is designed for simultaneous excitation at 458 and 514 nm. Autofluorescence emission could be observed in a broad wavelength range with a highly dominant peak at 670 nm.

Most substances contributing to autofluorescence background in plants share an excitation maximum in the violet/blue range, whereas the emission maxima are distinct between the fluorophores and, thus, affect spectral variants of fluorescent proteins to different extents (Roshchina, 2012; Table 2). Between these substances are secondary metabolites that accumulate in the vacuole, but also ubiquitously distributed molecules such as flavins. The main sources of autofluorescence are chlorophylls in the plant cell. Chlorophylls are characterized by a broad absorption and emission spectrum nearly affecting any fluorescent protein. The fluorescence emission spectrum of chloroplasts shows a prominent peak at 670 nm that corresponds to chlorophyll (Figure 6). However, chlorophylls are restricted to the thylakoid region of plastids so that their fluorescence is not critical for analyses in other compartments (Figure 6).

**Table 2:** **Sources of autofluorescence in plant cells**.

**Molecule**	**Localization**	**Emission wavelength**	**References**
Chlorophyll	Plastids	450–700 nm, 675–680 nm	Agati, 1998; Vitha and Osteryoung, 2011
**UV-VIOLET FLUORESCENT PROTEINS**			
Lignin	Cell wall	358 nm; 440 nm	Albinsson et al., 1999; Djikanović et al., 2007
**BLUE FLUORESCENT PROTEINS**			
Cellulose	Cell wall	420–430 nm	Pöhlker et al., 2011
NAD(P)H	Plastids	450 nm	Poot et al., 2002
Pterins/folates	Vacuole, mitochondria, plastids, cytosol	450 nm	Wolfbeis, 1985; Hossain et al., 2004
**CYAN FLUORESCENT PROTEINS–GREEN FLUORESCENT PROTEINS**			
Terpenes	(Flowers)	470–525 nm	Roshchina, 2012
Flavonoids	Ubiquitous	470–525 nm	Roshchina, 2012
Lipofuscin-like	(Pollen)	475–480 nm	Roshchina and Karnaukhov, 1999
**GREEN–YELLOW FLUORESCENT PROTEINS**			
Phenols	Vacuole, cell wall, chloroplasts	490–560 nm	Roshchina, 2012
Flavins	Ubiquitous	520 nm	Wolfbeis, 1985
Betaxanthins	Flowers	530–560 nm	Gandía-Herrero et al., 2005
β-carotin	Chloroplast, lipid globules	560 nm	Gillbro and Cogdell, 1989; Kleinegris et al., 2010
**YELLOW–ORANGE FLUORESCENT PROTEINS**			
Polyacetylene	Vacuole	530–595 nm	Roshchina, 2012
Isoquinoline	Vacuole	530–595 nm	Otani et al., 2005; Roshchina, 2012
Acridone alkaloids	Vacuole, (idioblasts)	530–595 nm	Eilert et al., 1986; Roshchina, 2012
**RED FLUORESCENT PROTEINS**			
Anthocyanins	Vacuole, (pollen)	600–630	Roshchina, 2012
Azulenes	(Pollen)	600–630	Roshchina, 2012

**Figure 7:**
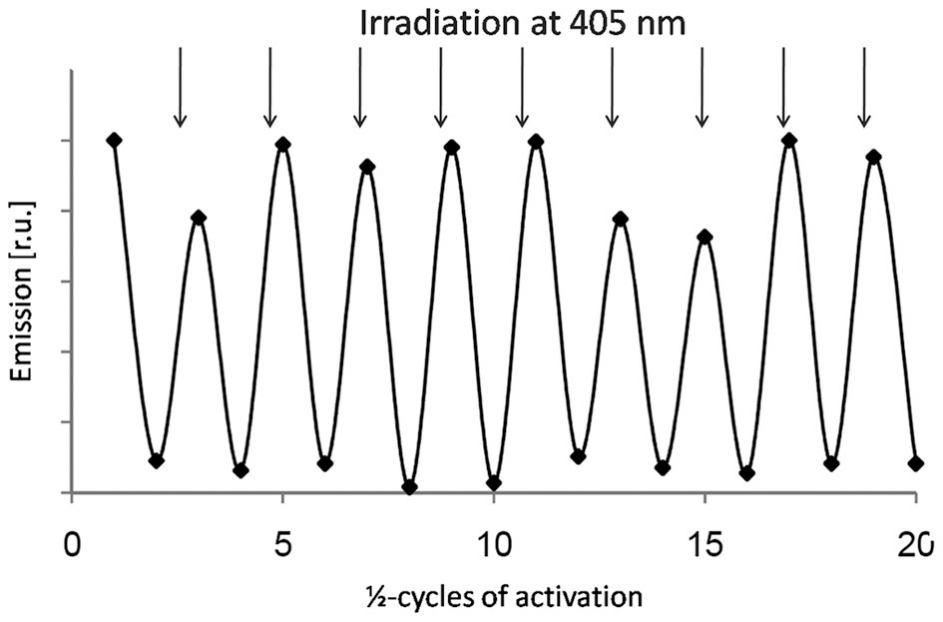
**Activation and inactivation cycles of Dronpa in plant cells.** Switching of Dronpa was analyzed by confocal laser scanning microscopy of Arabidopsis protoplasts. Emission was detected between 500 and 600 nm using excitation of 488 nm. Intensive illumination with 488 nm was performed to switch Dronpa off. Subsequent irradiation at 405 nm fully recovered the fluorescent state. The procedure was repeated for 10 times without significant loss in emission intensity.

**Figure 8:**
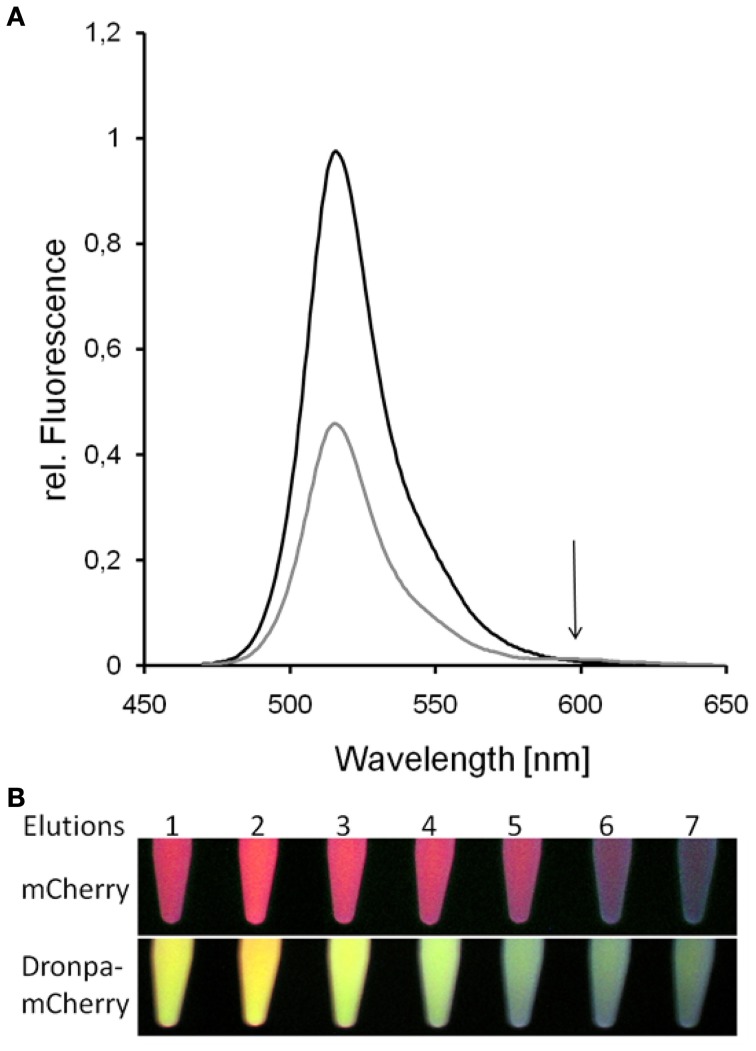
**Donor-quenching of Dronpa in the presence of mCherry.** The comparison of emission spectra of Dronpa and the fusion protein Dronpa-mCherry revealed the quenching of Dronpa in the presence of mCherry **(A)**. In the emission spectrum of Dronpa-mCherry the emission of mCherry is less pronounced due to the low quantum yield of mCherry. The same effect can be observed by the emission of eluted fluorescent proteins **(B)**: whereas mCherry showed red fluorescence, the emission of the fusion protein is dominated by the yellowish Dronpa fluorescence.

**Figure 9:**
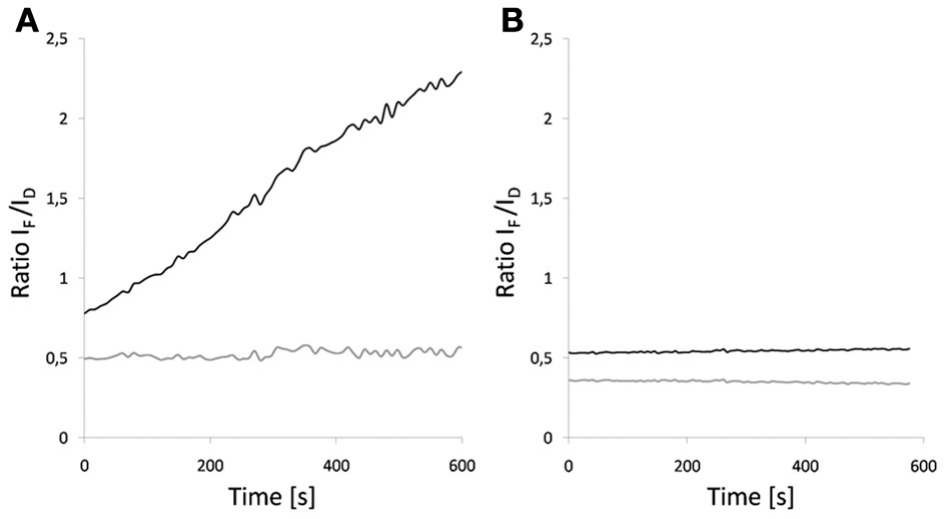
**Influence of photoswitching on FRET-ratio of a Dronpa-mCherry fusion.** Ratio imaging was performed as a time series. Images were obtained every 6–8 s for 10 min. **(A)** Dronpa was inactivated during the time series. **(B)** Dronpa was reactivated at the time points by irradiation with a 405 nm diode laser. The black line shows data that has not been corrected for acceptor spectral bleed through while the gray curve shows the corrected data.

Yellow and orange fluorescent proteins are characterized by a higher and more red-shifted spectral overlap with red fluorescent proteins. Accordingly, they enable high *R*_0_-values and thus a high dynamic distance range (Table 3; Goedhart et al., 2007; Akrap et al., 2010). However, Lam et al. (2012) suggested the green fluorescent protein Clover and the red fluorescent protein mRuby2 as ideal FRET-pair for expansion of the dynamic range due to their high *R*_0_ = 6.3 nm, absorption coefficients and quantum yields. Although the application of yellow donors is still in its infancy, these materials have a high potential for suppressing autofluorescence background, e.g., in plastids. Another interesting aspect is the development of photoswitchable green fluorescent proteins such as Dronpa for FRET. The emission spectrum of Dronpa shows significant overlap with the absorption spectrum of mCherry (Figure 2), resulting in an *R*_0_ = 5.58 nm. The advantage of this FRET-pair is the possibility to adjust the (active) donor to acceptor ratio. This permits, for example, identification of saturated acceptors in the presence of multiple donors and thus, improvement of donor to acceptor ratio, if donors form a homo-oligomer that interacts with a single acceptor. In 2-step FRET-cascades, photoswitchable mediators should enable to consider energy transfer from donor to acceptor that bypasses the mediator. On the other hand, photoswitchable donors provide a perspective for the combination of FRET and super resolution microscopy, if stability and reproducibility of switching is given. For Dronpa, the initial emission was fully recovered at least after 10 cycles of inactivation and activation (Figure 7). However, this number of repetitions is far from the number of repetitions required for photoactivation-dependent sub-diffraction microscopy. Moreover, donor persistence has to arise from single molecules rather than from ensembles. For an additional proof of concept, a fusion protein of Dronpa and mCherry was constructed and the intramolecular FRET-efficiency was determined as 0.53 (Figure 8). Next, ratio-imaging has been performed to analyse the robustness of FRET between both proteins while the donor fraction is gradually reduced in a time series. If ASBT is not considered, the ratio increases over time reflecting the increasing contribution of ASBT to the emission in the FRET-channel (Figure 9A), but subtraction of ASBT results in a stable ratio over time. In a second set of experiment, Dronpa was stabilized by irradiation with 405 nm before recording the emission at individual time points. In this case, the ratio was constant even if ABST was not considered and fluctuations were less pronounced than in the previous measurement (Figure 9B).

**Table 3:** **Förster-radii of fluorescent protein FRET-pairs**.

**Fluorophores**	**Förster radius *R*_0_**	**Dynamic range**	**References**
**BLUE DONOR**			
EBFP/ECFP	3.8 nm	1.9–5.7 nm	Patterson et al., 2000
EBFP/EGFP	4.1 nm	2.1–6.2 nm	Patterson et al., 2000
EBFP/EYFP	3.8 nm	1.9–5.7 nm	Patterson et al., 2000
EBFP/DsRed	3.2 nm	1.6–4.8 nm	Patterson et al., 2000
ECFP/EGFP	4.8 nm	2.4–7.2 nm	Patterson et al., 2000
ECFP/EYFP	4.9 nm	2.5–7.3 nm	Patterson et al., 2000
ECFP/mVenus	5.0 nm	2.5–7.5 nm	Rizzo et al., 2006
mCerulean/EYFP	5.4 nm	2.7–8.1 nm	Rizzo et al., 2006
mCerulean/mVenus	5.4 (5.2) nm	2.7–8.1 nm	Rizzo et al., 2006; Markwardt et al., 2011
mCerulean/mCitrine	5.4 nm	2.7–8.1 nm	Rizzo et al., 2006
mCerulean3/mVenus	5.7 nm	2.9–8.6 nm	Markwardt et al., 2011
SCFP3/SYFP2	5.4 nm	2.7–8.1 nm	Goedhart et al., 2007
mTurquoise/mVenus	5.7 nm	2.9–8.6 nm	Markwardt et al., 2011
mTurquoise2/mVenus	5.8 nm	2.9–8.7 nm	Goedhart et al., 2012
ECFP/DsRed	4.2 (5.1) nm	2.1–6.3 nm (2.6–7.7 nm)	Patterson et al., 2000; Erickson et al., 2003
ECFP/mCherry	3.5 nm	1.8–5.3 nm	He et al., 2005
EGFP/EYFP	5.6 nm	2.8–8.4 nm	Patterson et al., 2000
EGFP/DsRed	4.7 (5.8) nm	2.4–7.1 nm (2.9–8.7 nm)	Erickson et al., 2003
EGFP/mRFP1	4.7 nm	2.4–7.1 nm	Peter et al., 2005
Clover/mRuby2	6.3 nm	3.2–9.5 nm	Lam et al., 2012
Kaede/Kaede	5.8 nm	2.9–8.7 nm	Wolf et al., 2013a
Dronpa/mCherry	5.6 nm	2.8–8.4 nm	This work
EYFP/DsRed	4.9 nm	2.5–7.4 nm	Patterson et al., 2000
EYFP/mCherry	5.7 nm	2.9–8.6 nm	Akrap et al., 2010
SYFP2/mStrawberry	6.3 nm	3.2–9.5 nm	Goedhart et al., 2007
mKo/mCherry	6.4 nm	3.2–9.6 nm	Goedhart et al., 2007

Recently, the photoconvertible fluorescent protein Kaede was used as both donor and acceptor for analysis of homo-oligomerization and conformational alterations. Kaede can be irreversibly converted from a green form to a red form by irradiation at 405 nm. Both forms show high degree of spectral overlap and thus a high *R*_0_ = 5.74 nm (Wolf et al., 2013a). The green form of Kaede was gradually converted to the red form and donor-, acceptor- as well as FRET-images (see 3-filter FRET) were obtained while conversion was in progress (Wolf et al., 2013a). Thus, the application of Kaede for the generation of transgenic plants enables multiple analyses in plants ranging from localization experiments with improved separation from autofluorescence, observation of protein dynamics and last but not least monitoring aggregation or conformational dynamics via FRET (Wolf et al., 2013a).

## Application of FRET in living cells

### Nanosensors

FRET-based nanosensors are synthetic constructs that share high similarity in their structural design. Usually fluorescent proteins are applied as donor and acceptor that are linked by a sensing domain. Upon binding of the respective ligand the sensing domain undergoes a conformational alteration that can be read out as alteration of FRET-derived emission. Here, the dynamic range of a sensor was defined as range of FRET-efficiency over which the sensor operates (Lam et al., 2012). The function of the sensors might be sensitive to ionic strength and pH, development and application of sensors of low and high affinity for the ligand discriminates between unwanted environmental effects and true sensor response (Chaudhuri et al., 2011). The most prominent sensors are the Ca^2+^-sensor of the cameleon-type (Miyawaki et al., 1997). Their sensing domain consists of calmodulin and the calmodulin-binding domain M13. Ca^2+^-binding results in a conformational alteration that favors FRET (Nagai et al., 2004). Although cameleons cause calcium-buffering in the cell, the interference with endogenous calmodulin and M13-domains is negligible (Miyawaki et al., 1999). The frequently applied cameleon YC3.6 represents a low affinity variant and consists of ECFPΔC11 and cpVenus which are linked by calmodulin and the calmodulin-binding M13-domain. Recently, compartment specific variants of YC3.6 were constructed and successfully expressed in plant cells (Krebs et al., 2012). A comprehensive overview of available sensors and their ground-lying design, properties, and limitations has been summarized in Okumoto et al. (2012).

### Conformational alterations

During their life time proteins principally undergo multiple structural alterations beginning with initial folding, posttranslational modification in terms of regulation and finally degradation. Intramolecular FRET can be applied to visualize protein folding or degradation, if the tertiary structure of the protein positions its termini in a way allowing for FRET in the fully folded mature protein. Structural flexibility of the unfolded conformation or proteolytic processing due to degradation would result in loss of FRET as it has been reported for *Escherichia coli* proteins (Philipps et al., 2003).

Proteolytic activation of the membrane bound transcription factor ANAC089 was observed in *A. thaliana*. To this end, ECFP was fused to its C-terminus, labeling the membrane integral domain, and EYFP to its N-terminus, labelling the cytosolic domain. Under reducing conditions loss of FRET demonstrated the release of the cytosolic domain that subsequently translocated to the nucleus (Klein et al., 2012).

Alterations of the redox environment affect the conformation of many proteins, e.g., by post translational modifications such as disulfide formation. The structure of the typical 2-cysteine peroxiredoxin was found to be responsive to alterations in the plastidic redox state as two populations of FRET-efficiencies were observed that correspond to the reduced and the oxidized state, respectively, and the protein reacted reversibly to the supply of reduced dithiothreitol (DTT) or hydrogenperoxide. Similar to the previously described constructs, 2-Cys Prx was fused to ECFP at its N-terminus, while the transit peptide was maintained at the extreme N-terminus, and EYFP was fused to its C-terminus (Muthuramalingam et al., 2009). Conformational changes can also be observed in larger complexes such as the 800 kDa vacuolar H^+^-ATPase. ATP-depletion by deoxyglucose-supply resulted in a movement of the cytosolic sector V_1_ relative to the membrane integral sector V_0_ and an altered arrangement of peripheral subunits within the cytosolic V_1_-sector (Schnitzer et al., 2011).

### Protein-protein-interactions

Complex formation is a common feature of many proteins for forming holo-enzymes, cooperative motifs or microenvironments for a successive sequence of reactions. On the other hand, protein-protein interactions are often involved in protein regulation, either in a short-term or in a long-term manner. FRET enables the visualization and quantitative analysis of protein-protein interactions between at least two proteins, if a protein is genetically fused to the donor and its putative interaction partner to the acceptor. By doing so, many proteins have been analyzed for interaction, just some recent examples are listed: (i) phosphorylation-dependent homo-dimerization has been detected for the response regulator ARR18 (Veerabagu et al., 2012), whereas (ii) GAGA-binding factors BBR/BPC dimerize constitutively in nucleus and nucleolus (Wanke et al., 2011). (iii) Competitive binding of flavonol synthase 1 to chalcone synthase and dihydroflavonol-4-reductase has been demonstrated in *A. thaliana* (Crosby et al., 2011). (iv) The mitochondrial serine acetyltransferase interacts reversibly with O-acetylserine (thiol) lyase to regulate the sulfur homeostasis in tobacco (Wirtz et al., 2012).

## Measuring FRET in living plant cells

### Methods for FRET-analysis—an overview

In the last decade several methods and experimental setups were applied for the analysis of FRET in living cells that rely on the property of RET to affect the excited state of donor and acceptor. Comparatively monitoring the donor's fluorescence or fluorescence lifetime in absence and presence of the acceptor, recording the acceptor's emission due to FRET and analysis of donor anisotropy were recruited for the analysis and quantification of FRET. In all cases the obtained FRET-efficiency is a function of energy transfer between donor and acceptor and of the donor fraction taking part in complex formation with acceptors (Xia and Liu, 2001). It turned out that different methods gave results that correlated quite well by tendency, but the exact values differed (Domingo et al., 2007).

### Acceptor bleaching

**Figure 10:**
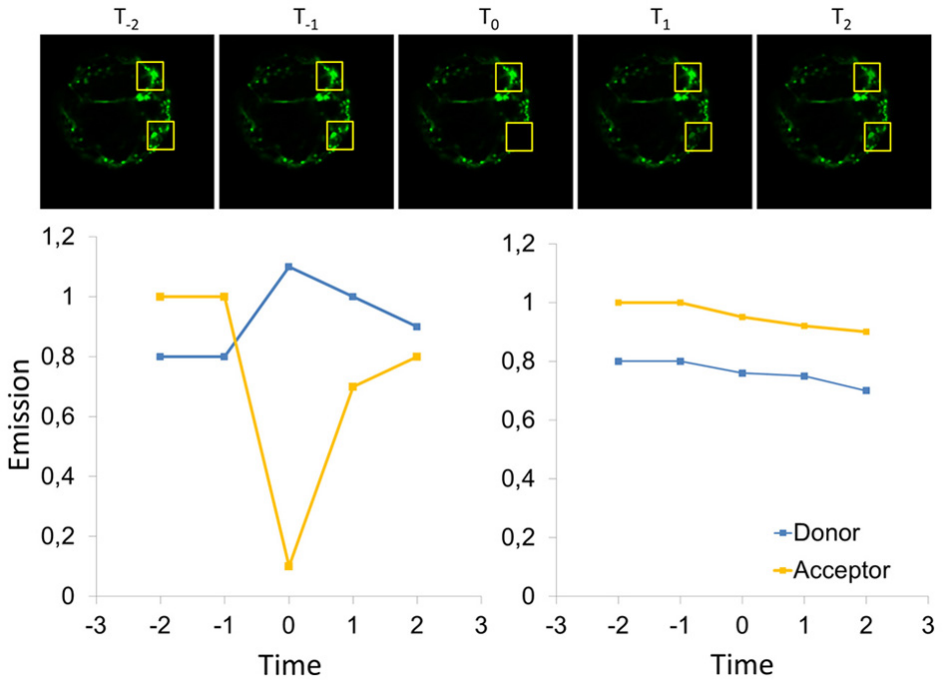
**Scheme for FRET-measurement by acceptor bleaching.** For determination of the FRET-efficiency by acceptor bleaching, the acceptor is bleached in a defined area of the cell before time point T_0_, and the emission of donor and acceptor is recorded subsequent bleaching. In addition, a region of interest is considered that has not been bleached (upper row) and allows for correction of fluctuations of the cellular fluorescence, e.g., it is expected that some bleaching occurs in the entire cells (right panel). In the bleached area, the acceptor emission decreases and the emission of the donor increases since FRET does not contribute to relaxation of the donor anymore. At later time points (T_1_ and T_2_) fluorescence recovery might be observable depending on the diffusional properties of the analyzed proteins, so that acceptor bleaching connects FRET to mobility measurements by fluorescence recovery after photobleaching (FRAP). Accordingly, highly mobile proteins prevent FRET-measurements by acceptor bleaching, if the recovery is faster than image acquisition subsequent bleaching.

The donor transfers energy to the acceptor so that the donor emission is quenched. Upon intensive or prolonged irradiation of the acceptor the fluorophore becomes irreversible inactivated and emission of the donor is recovered. The advantage of this method is the reproducibility of obtained FRET-efficiencies independent of the experimental setup. In 2003, a comprehensive and reliable procedure was suggested for determining FRET by acceptor bleaching (Figure 10). The protocol involves positive controls such as donor-acceptor fusions, negative controls such as donor only and analysis of non-bleached regions of interest (Karpova et al., 2003). A fundamental prerequisite is the correction for donor bleaching that might occur in parallel with acceptor bleaching (Daelemans et al., 2004; van Munster et al., 2005). Therefore, the combination of less stable acceptor and a stable donor is favorable (Bhat, 2009). Problematic is the application of acceptor bleaching in living cells. Acceptor bleaching by a laser usually takes ~1 min, therefore exclusively fixed cells or immobile proteins can be analyzed (Piston and Kremer, 2007). The attempt to overcome the long exposure by high light intensities is accompanied by high photo-toxicity (Xia and Liu, 2001). In particular highly pigmented cells suffer from high level of irradiation. Also, the abundance of fluorophores contributes to photo-toxicity (Dixit and Cyr, 2003). Another possibility that has been developed for conventional fluorescence microscopy relies on gradual acceptor bleaching and fit of the decay curve (van Munster et al., 2005). However, FRET is a mechanism of relaxation of excited molecules that represents a change in the electronic environment of the donor. Living cells and fixed differ in the environment of the fluorophores, resulting in distinct behavior of fluorophores. Photostability of CFP decreased in fixed cells while Venus showed increased stability. On the other hand, the photostability of mTFP1 and Cerulean increased subsequent fixation whereas YFP was unaffected (Malkani and Schmid, 2011). This indicates that donors such as mTFP1 and Cerulean are suitable for acceptor bleaching in fixed cells in combination with Venus as acceptor. Additional drawbacks are the tendency of fluorescent proteins to undergo photoconversion that is hard to separate from photobleaching (Kremers and Goedhart, 2009), potential changes in cell morphology and focal position (Zal and Gascoigne, 2004) as well as the incomplete bleaching in particular in living cells (Zal and Gascoigne, 2004; Wallrabe and Periasamy, 2005), although corrections were provided for incomplete bleaching (Dinant et al., 2008). In detail, the commonly applied fluorescent protein FRET-couple CFP/YFP suffers from photoconversion of YFP to a cyan form especially in fixed cells putatively due to dehydration and reduced heat dissipation (Valentin et al., 2005; Raarup et al., 2009). On the other hand, photoactivation of CFP and Cerulean has been reported upon bleaching of YFP so that increase of cyan emission is not restricted to the absence of the acceptor but contains a photoactivation-related portion with hard to determine contribution (Malkani and Schmid, 2011). Nevertheless, acceptor bleaching has been reported to be more precise than FLIM-FRET in the case of multi-exponential decaying donors (Goedhart et al., 2007). Accordingly, discrepancies among results from acceptor bleaching and FRET-FLIM were reported for the FRET-pair CFP/YFP due to a four component exponential decay curve (Vermeer et al., 2004). Rarely, acceptor bleaching experiments were followed by fluorescence recovery after photobleaching (FRAP) so that diffusion of intact acceptors into the bleached area resulted in recovery of donor quenching and gave insights into the mobility of the acceptor (Vermeer et al., 2004).

### Donor bleaching

The detection of donor bleaching allows for qualitative analysis of FRET and relies on the increased stability of the donor in presence of an acceptor. Hence the bleaching constant increases and it takes longer to bleach the donor completely (Schmid et al., 2001; Daelemans et al., 2004; Szentesi et al., 2005). The time constant of donor bleaching is inversely related to the quantum yield of the donor (Jares-Erijman and Jovin, 2003). The donor decay curve is bimodal due to the fraction of donor that is associated with the acceptors and the fraction that emits directly (Clayton et al., 2005). Donor bleaching experiments require a labile donor and photo stable acceptor that is not necessarily fluorescent, and allow for the quantification of the donor fraction that transfers its energy to the acceptor (Jares-Erijman and Jovin, 2003; Clayton et al., 2005). According to acceptor bleaching, recording the complete bleaching curve might be critical in living cells since morphology and fluorophore distribution may vary at different time points, but data evaluation can be performed based on single steps of bleaching (Clayton et al., 2005).

### Fluorescence lifetime of the donor

The fluorescence lifetime of a fluorophore depends on the electronic nano environment so that fluorescence lifetime imaging microscopy (FLIM) is suitable for the analysis of local environmental conditions and can be used for the detection of interactions between proteins and lipids or DNA, respectively (Lakowicz, 2006). If an appropriate acceptor is close to a potential donor, the fluorescence lifetime of the fluorophore decreases due to FRET to the acceptor. Based on the difference in lifetime the FRET-efficiency can be calculated:

(5) $$E=1-\frac{\tau_{da}}{\tau_{d}}$$

**Figure 11:**
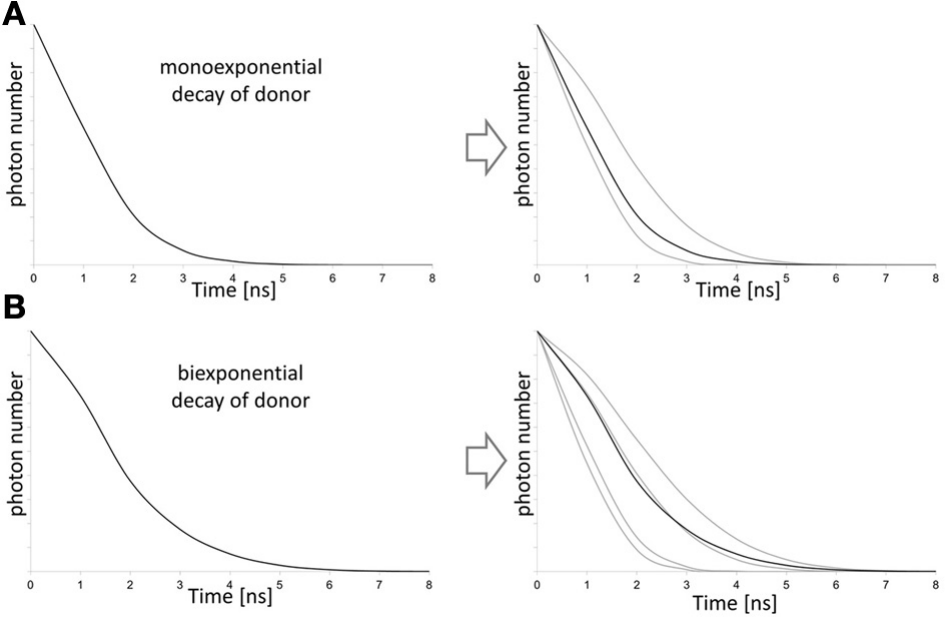
**Influence of the donor decay function on FLIM-FRET evaluation.** On the left mixtures of decays (black curve) are shown that are separated in the lifetime decays (gray curves) of the contributing states of the putative donor on the right. For donor fluorophores that show a monoexponential fluorescence decay, a bi-exponential fit is required for data evaluation to consider donor fraction that transfer energy to the acceptor and the donor fraction that emits directly **(A)**. If the donor already shows a biexponential behavior fluorescence decay behavior, four contributing fractions of donor have to be considered for the fit of the lifetime decay **(B)**. The fit also provides information on the fraction of donor that undergoes energy transfer to the acceptor.

Whereas the nano environment is equal for all donor molecules and result in an identical electronic state and thus monoexponential decay, the presence of acceptors leads to a fraction of donors that undergo FRET and donors that do not. This results in a bi-exponential decay curve that has to be fitted for two species (Figure 11; Padilla-Parra et al., 2008). Thus, FLIM depends on the decay curve and its exponential behavior to separate interacting and non-interacting donors (Duncan, 2006). The fraction of donor showing FRET relies on the photostability of the acceptor (Padilla-Parra et al., 2009). Two approaches are routinely used for the measurement of the donor fluorescence lifetime: (i) Time domain FLIM depends on a pulsed laser source and time-gated detectors those allow for the observation of the time point of emission (Gerritsen et al., 2009). (ii) On the other hand frequency domain method relies on modulation of the frequency of the excitation light and modulated detection (Verveer and Hanley, 2009). Unfortunately, the comparison of lifetimes gained by different methods may be hampered by distinct fit-algorithms (Padilla-Parra et al., 2008). Time-correlated single photon counting (TCSPC) is commonly applied on confocal laser scanning microscopes. Here, photons are counted over a defined time interval in a pixelwise manner so that measurements of the fluorescence lifetime in the nucleus were reported to take 3 min. During this time morphological changes in the cell and dynamics in protein localization are likely (Piston and Kremer, 2007; Padilla-Parra et al., 2008). The limitation in speed can potentially be overcome by reduced image resolution and analyzing small regions of interest (Duncan, 2006). Time-gated detection and frequency-domain FLIM in combination with spinning disc microscopy enable high imaging speed (Domingo et al., 2007; Padilla-Parra et al., 2008). However, TCSPC has the advantage of comparatively low excitation light and hence less impact on the cells and less bleaching of the donor (Tramier et al., 2006).

Besides the requirement for specific experimental setups, the usage in the living cell is partially hampered by the requirement for fluorophores with monoexponential fluorescence decay, so that the commonly used FRET-pair ECFP/EYFP is less suitable for FLIM-FRET. CFP undergoes photoconversion leading to multiple lifetime species (Figure 11; Tramier et al., 2006). In particular appropriate fluorescent proteins are EGFP that is characterized by insensitivity to photobleaching, mTFP1 with high photostability, AmCyan that unfortunately aggregates, (Padilla-Parra et al., 2009), T-Sapphire (Bhat, 2009), Cerulean (Duncan, 2006), and mKO (Kremers and Goedhart, 2009), SCFP3A and SYFP2 (Kremers et al., 2006). On the other hand, blinking of the acceptor results in multiple lifetime states of the donor and affects data evaluation (Vogel et al., 2012). Also, long lived-acceptors may become saturated by FRET so that acceptors of short fluorescence lifetime are of advantage not exclusively for FLIM-FRET but also for FRET in general (Jares-Erijman and Jovin, 2003). For the donor, a long lifetime increases the probability of FRET (Vogel et al., 2012). Since GFP and YFP represent a highly efficient FRET-couple but with the difficulty of spectral separation (Dinant et al., 2008), non-fluorescent acceptors were designed based on YFP to improve the spectral overlap of donor emission and acceptor absorption without the need for spectral separation of the emission. This resulted in the non-fluorescent variants REACh, REACh2, and sREACh of EYFP (Ganesan et al., 2006; Murakoshi et al., 2008). These proteins serve as donors for EGFP. The residual fluorescence of these proteins is less than 3% of the YFP-fluorescence. However, if concentration is high e.g., in proteasomes, REACh's fluorescence might be detectable (Ganesan et al., 2006).

### Spectral imaging

Confocal laser scanning microscope allow for recording emission spectra, either by stepwise recording of the emission or by simultaneous detection with an array of detectors or sections of a CCD-chip (Rizzo et al., 2006; Megías et al., 2009). These measurements were termed fluorescence spectral imaging microscopy (FSPIM; Vermeer et al., 2004). The stepwise recording suffers from low acquisition speed as it takes several seconds to obtain a spectrum (Megías et al., 2009). For the detection of FRET at least one spectrum is recorded that covers the emission of both donor and acceptor upon excitation of the donor. A second spectrum covering the acceptor emission upon its excitation provides information on the acceptor abundance, since acceptor signal and FRET-signal are identical in shape and cannot be separated by linear unmixing (Chen et al., 2007). Spectral imaging was reported to be insensitive to autofluorescence and high degree of spectral overlap since the contribution of individual fluorophores can be separated (Megías et al., 2009). In the past, spectral imaging has been combined with acceptor bleaching resulting in a long-lasting procedure that appears not applicable for analysis with subcellular resolution or mobile cytosolic proteins (Kluge et al., 2004; Raicu et al., 2005).

### Detecting sensitized emission by ratio-imaging

Ratio imaging represents the simplest approach to observe FRET since only two channels are required. The emission of the donor *I*_*D*_ is recorded in the first channel, the FRET-derived emission of the acceptor *I*_*F*_ upon donor excitation in the second channel. FRET results in decreased donor emission and increased acceptor emission so that the ratio *I*_*D*_/*I*_*F*_ decreases (Miyawaki and Tsien, 2000). Typically, the ratio *R*_FRET_ of the emissions in the resulting channels is calculated. Neither donor-crosstalk nor direct acceptor excitation are considered:

(6) $$R_{\text{FRET}}=\frac{I_{F}}{I_{D}}$$

Calculating the ratio gives consistent values with less variation, but normalization to donor and acceptor expression level is not included (Xia and Liu, 2001). Therefore, the method depends strongly on the ratio of donor and acceptor and is exclusively suitable for monitoring intramolecular FRET so that donor and acceptor ratio is constant and known (Gordon et al., 1998; Domingo et al., 2007). Thus, it is frequently applied for FRET-analysis of FRET-sensors that consists of donor and acceptor linked by a sensing peptide.

### 3-filter FRET (sensitized emission)

**Figure 12:**
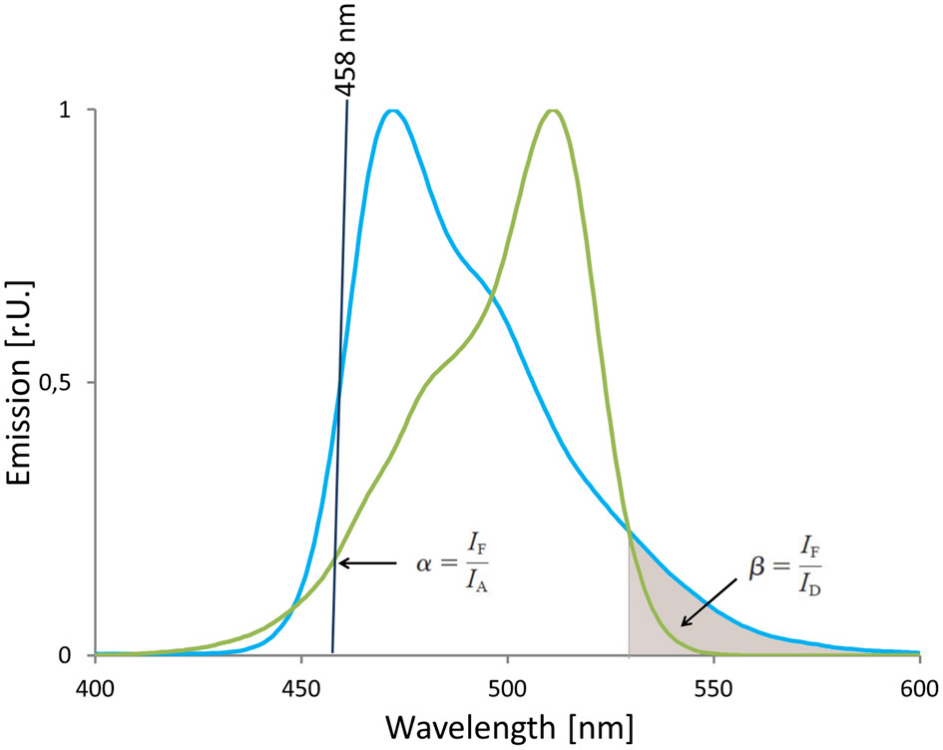
**Estimation of correction factors for donor cross talk and acceptor's direct excitation.** The absorption spectrum of mVenus and the emission spectrum of mTurquoise2 are shown. The 458 nm laser line (black line) that is used for excitation of mTurquoise2 is in the wavelength range that is covered by the absorption spectrum of mVenus, too. On the other hand, the emission spectrum of mTurquoise2 overlaps with the emission spectrum of mVenus and is also detected in the FRET-channel as indicated by the gray area below the mTurquoise2 emission spectrum. Thus, the factors α and β correct for acceptor's direct excitation at donor's excitation wave length and donor crosstalk into the FRET-channel. The correction factors α and β are calculated based on data sets from cells that express solely mVenus or mTurquoise2, respectively.

The main drawback of ratio-imaging is the inability to correct for variations in donor to acceptor ratio and for ASBT that is caused by direct excitation at the donor excitation wavelength. Supplementing the ratio imaging by a third channel that detects the fluorescence emission of the acceptor *I*_*A*_ upon acceptor excitation enables for the quantification of the fraction of detected emission that is exclusively related to FRET. The direct excitation of acceptor or ASBT is linearly related to the emission intensity *I*_*A*_ detected in the acceptor channel and linearity is described by the proportionality factor α. The relative amount of donor spectral bleed through (DBST) depends on detector settings and the detection range and is given by the correction factor β (van Rheenen et al., 2004). Both correction factors can be determined experimentally applying cells that express solely donor (Equation 8 for determination of β, Figure 12) or acceptor (Equation 7 for determination of α, Figure 12). Hoppe et al. (2002) estimated α and β with recombinant purified protein besides the determination in living cells. The obtained values were reported to be in good agreement with data derived from cells expressing the fluorescent proteins.

(7) $$\alpha =\frac{I_{F}}{I_{A}}$$

(8) $$\beta =\frac{I_{F}}{I_{D}}$$

Finally, the FRET-derived emission intensity *I*_corr_ is given by Equation 9:

(9) $$I_{\text{corr}}=I_{F}-\alpha I_{A}-\beta I_{D}$$

Several calculations can be found in the literature that are linked to the FRET-efficiency but mostly give values that are linear related to and correlate well with the FRET-efficiency but do not match it exactly. In general, acceptor-related and donor-related equations can be distinguished for calculating apparent FRET-efficiencies, depending on if acceptor emission or donor emission contributes to the nominator (van Rheenen et al., 2004). Most acceptor-based equations are highly sensitive to the detection of acceptor's emission and its capability to be excited at donor's excitation wavelength. The simplest equation relies on relating the FRET-signal to the acceptor emission and is robust to a lack of ABST (Domingo et al., 2007).

(10) $$E=\frac{I_{F}-\beta I_{D}-\alpha I_{A}}{I_{A}}$$

This equation might be applicable for conventional fluorescence microscopy, but critical with a confocal laser scanning microscope that offers the possibility to adjust excitation intensities independently. Alternatively, the acceptor emission in presence of donor can be related to acceptor emission in absence of the donor so that differences in excitation intensities are considered by α (Wolf et al., 2013a):

(11) $$E=\left(\frac{I_{F}-\beta I_{D}}{\alpha I_{A}}-1\right)$$

Here, significant ASBT of acceptor is strictly required and the correction factor α depends on the laser intensity ratio of donor and acceptor excitation (van Rheenen et al., 2004). If ASBT is absent, the nominator becomes 0 and thus undefined.

Donor-based quantifications seem to be more robust to variations of the correction factors. In a simple way, apparent FRET-efficiency can be expressed as FRET-derived emission intensity *I*_corr_ related to the sum of *I*_corr_ and *I*_*D*_. This relies on the assumption that the donor is quenched if FRET occurs, and the sum of *I*_corr_ and *I*_*D*_ is linearly related to the unquenched donor emission (Seidel et al., 2005; Schnitzer et al., 2011).

(12) $$E=\frac{I_{F}-\beta I_{D}-\alpha I_{A}}{\left(I_{F}-\beta I_{D}-\alpha I_{A}\right)+I_{D}}$$

In another approach, normalization to both donor and acceptor was applied to reduce the variation of filter FRET-measurements. This was achieved by calculating the square root of the product of donor and acceptor emission and normalizing *I*_corr_ to the obtained value (Xia and Liu, 2001):

(13) $$E=\frac{I_{F}-\beta I_{D}-\alpha I_{A}}{\sqrt{I_{D}I_{A}}}$$

However, all these equations result in apparent FRET-efficiencies at least qualitatively proving a protein-protein interaction, since its relationship to the true FRET-efficiency is not known due to the lack of calibration of the experimental setup and correction for the cellular environment. Expression of freely diffusing donors and acceptors has been suggested as negative control for occasionally occurring FRET between fluorescent protein donors and acceptors, in particular under conditions of overexpression (Xia and Liu, 2001; Erickson et al., 2003). The obtained apparent FRET-efficiency represents a threshold for accepting the hypothesis of interaction that can be verified by statistical analysis. Overexpression is known to cause spurious FRET that is enhanced by aggregating fluorescent proteins (Erickson et al., 2003). Thus, the avoidance of highly expressing cells further reduces the probability of unspecific interaction (Xia and Liu, 2001). Nevertheless, the apparent FRET-efficiencies are suitable for monitoring conformational dynamics inside complexes and *de novo* complex formation or complex decomposition as long as the structural alterations result in a significant shift of fluorophore's distance or relative orientation.

## Calibration and quantification procedures for filter FRET

There are several reasons for calibrating FRET-data. The obtained data vary in the FRET-efficiency due to the application of FRET-pairs with distinct R_0_ and thus, different dynamical range, heterogeneity of experimental setups and experimental procedures and variety of methods for data evaluation. Comparing data obtained with different FRET-pairs is quite simply enabled, if FRET-efficiencies are expressed as their corresponding distances so that *R*_0_ is considered and used to normalize FRET-measurements performed with distinct FRET-pairs, even though the calculation of distances is principally incorrect due to lack of information on the chromophores' orientation:

(14) $$R=R_{0}\sqrt[{6}]{\frac{1}{E}-1}$$

**Figure 13:**
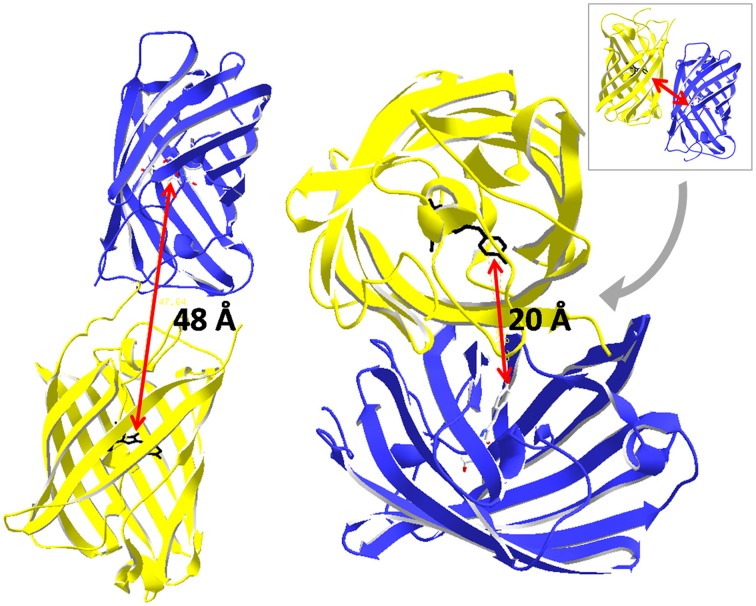
**Approximation of nearest neighbor distances between fluorescent proteins.** The fluorophore is located on the central α-helix inside the β-can structure. Thus, the fluorophores of two fluorescent proteins are separated by at least 30 Å. Based on the crystal structure of a GFP dimer (pdb 1 GFL, Yang et al., 1996) the distance is 20 Å. The distance was 48 Å in a head to tail arrangement as it is most probably the case for fusion proteins that are frequently applied for FRET standardization.

**Figure 14:**
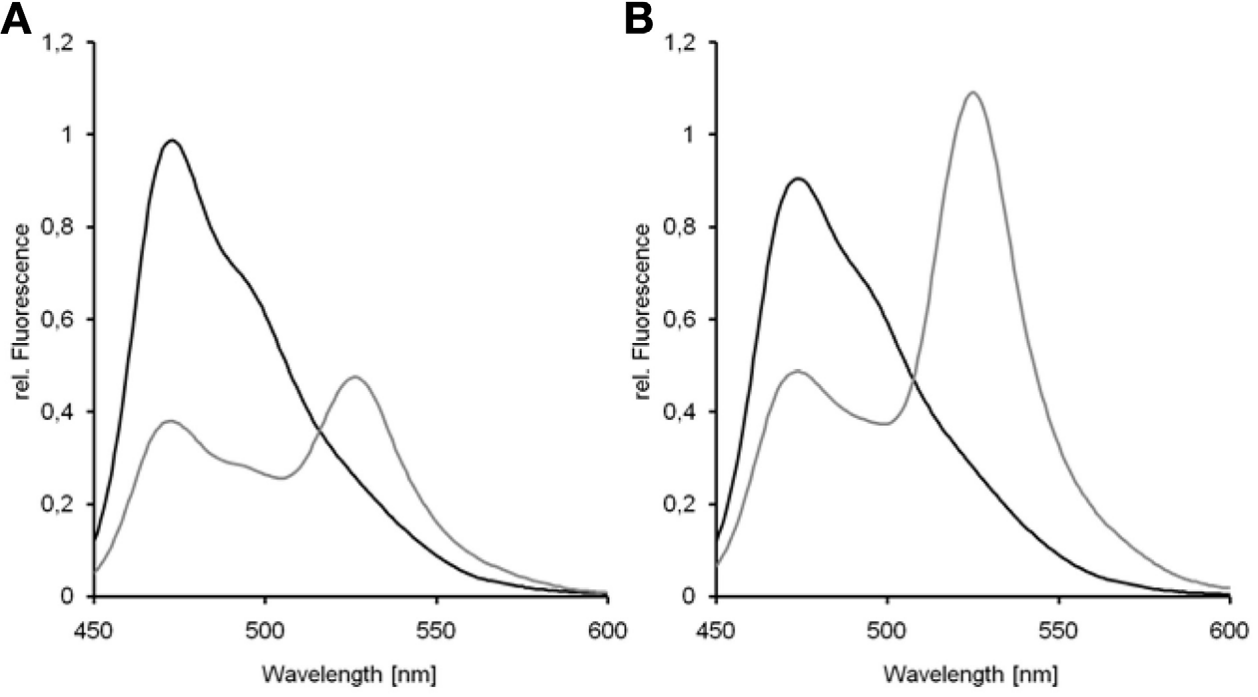
**Donor-quenching of ECFP and mTurquoise2 in the presence of an acceptor.** Emission spectra of donor only (black) and donor-acceptor fusion (gray) were recorded with a fluorescence spectrometer. Donor quenching of mTurquoise2 was higher in the presence of mVenus **(A)** than the quenching of ECFP in the presence of EYFP **(B)**.

The detection of donor and acceptor signals allows for crosstalk and direct excitation correction of both fluorophores and enables relative FRET-measurements to draw conclusions on structural alterations, but the obtained values are widely irreproducible with other experimental setups. The emission depends strongly on excitation intensity, detector sensitivity, and donor's and acceptor's concentration (Jalink and van Rheenen, 2009). Therefore, several corrections were suggested to consider different quantum yields and absorption coefficients of donor and acceptor, spectral transmission of required filters, and fluctuations in excitation intensities (Gordon et al., 1998; Hoppe et al., 2002; van Munster et al., 2005; Beemiller et al., 2006; Chen et al., 2006). The formulas have to be robust even if the stoichiometry and subcellular microenvironment of fluorescent proteins is unknown (Jares-Erijman and Jovin, 2003). Most of the corrections involved standardization of FRET by e.g., expression of reference constructs of known FRET-efficiency and fluorophore stoichiometry (Hoppe et al., 2002; Zal and Gascoigne, 2004; Beemiller et al., 2006; Chen et al., 2006) or gradual acceptor bleaching (van Munster et al., 2005). Typically, the reference constructs consists of donor and acceptor linked by a short amino acid sequence ranging from 5 to 32 amino acids. A possible arrangement of such a reference construct in comparison to the native GFP-dimer is given in Figure 13. In particular for the construction of cyan and YFP reference constructs linkers of 5, 17, and 32 amino acids were frequently applied (Koushik et al., 2006; Chen et al., 2007; Megías et al., 2009). Based on a length of 2.8 Å per amino acid this corresponds to linkers of 1.4–8.96 nm. Since the linkers do not possess a rigid secondary structure, the flexibility increases with increasing linker length so that the relative orientation of the fluorophores becomes highly variable and is more and more influencing and limiting FRET. This increasing complexity has to be considered if calibration is based on reference constructs of high linker length. Furthermore, fluorescent proteins are characterized by heterogeneity such as wide differences in their maturation. Whereas mCherry has a maturation-half time of 15 min its wildtype DsRed needs more than 10 h to maturate (Shaner et al., 2004) and the temperature optima of maturation differs e.g., between GFP and DsRed (Mizuno et al., 2001). Last but not least, different photo-stabilities of donor and acceptor, sensitivity to the environment, or combination of short lived donors with long lived acceptors have to affect intramolecular FRET within reference constructs (Padilla-Parra et al., 2009; Vogel et al., 2012). All these are reasonable explanations for variations in the spectroscopic behavior of donor-acceptor-fusions that result in e.g., an apparent deviation from the expected amount of donor undergoing FRET (Padilla-Parra et al., 2009). For obtaining the true FRET-efficiency of reference constructs by FLIM, Koushik et al. (2006) suggested fusing the non-fluorescent β-can protein Amber to the donor for recording its lifetime in the absence of the acceptor, since fusion proteins showed a distinct fluorescence lifetime than donor only. Comparing reference constructs for the FRET-pairs ECFP/EYFP and mTurquoise2/mVenus demonstrated the influence of higher quantum yield on the *R*_0_ and thus on the FRET-efficiency (Figure 14). The reduction in donor emission is significantly higher for mTurquoise2 in the presence of mVenus (*E* = 0.62, Figure 14A) than for ECFP in the presence of EYFP (*E* = 0.46, Figure 14B). If tandem fluorophores such as tdTomato are applied as acceptors, the more complex situation for the fluorophores' spatial arrangement and energy transfer pathways has to be considered, although the presence of multiple acceptors for a single donor represent a way of improving resonance energy transfer, since the operational *R*_0_ increases with the number of acceptors (Jares-Erijman and Jovin, 2003). The presence of two acceptors has also an effect on the FRET-efficiency: if Cerulean was fused to two copies of Venus, the FRET-efficiency increased from 0.45 to 0.6 (Chen et al., 2007; Koushik et al., 2009).

Most calculations and calibrations rely on the linear relationship of donor quenching and sensitized acceptor emission (Gordon et al., 1998; Hoppe et al., 2002; Zal and Gascoigne, 2004; Chen et al., 2006). The required proportionality factor has been termed G (or ξ) and depends in technical terms on the quantum yields Φ of donor and acceptor, the transmission Filter_trans_ of the filter sets for donor- and acceptor-detection and the coverage Filter_fract trans_ of the emission spectra by the filter transmission that is given by the integral of the product of the fluorescence emission spectrum and the transmission spectrum related to the integral of the fluorescence emission spectrum (Gordon et al., 1998):

(15) $$G=\frac{\phi_{A}}{\phi_{D}}\frac{{\text{AccFilter}}_{\text{trans}}}{{\text{DonFilter}}_{\text{trans}}}\frac{{\text{FRETFilter}}_{\text{fracttrans}}}{{\text{DonFilter}}_{\text{fracttrans}}}$$

For confocal laser scanning microscopy, an equivalent factor was suggested that is defined by the ratio of the quantum yields of acceptor and donor as well as the ratio of the quantum yields of the photomultipliers at the acceptor's and donor's wavelength (Sun and Periasamy, 2010). Assuming the proper calibration of the detectors with respect to their wavelength dependency at least in a narrow range, the calculation of the coefficient is limited to the ratio of the quantum yields of acceptor and donor. By doing so, the coefficient for e.g., EYFP and ECFP would be 0.66. Finally, the FRET-efficiency is given by Equation 16:

(16) $$E=\frac{\text{coef }\left(I_{F}-\alpha I_{A}-\beta I_{D}\right)}{\text{coef }\left(I_{F}-\alpha I_{A}-\beta I_{D}\right)+I_{D}}$$

However, the factor *G* can be experimentally determined either by photobleaching of the acceptor or by applying reference constructs. FRET-correction by photobleaching involves two measurements of sensitized emission. The first is performed before acceptor bleaching whereas the second set of images is obtained after acceptor bleaching. The difference of the *I*_corr_-values is divided by the difference of the emission in the FRET-channel after bleaching *I*^post^_*F*_ and the emission in the donor channel before bleaching *I*_*D*_ (Zal and Gascoigne, 2004):

(17) $$G=\frac{\left(I_{F}-\alpha I_{A}-\beta I_{D}\right)-\left(I_{F}^{\text{post}}-\alpha I_{A}^{\text{post}}-\beta I_{D}^{\text{post}}\right)}{I_{F}^{\text{post}}-I_{D}}$$

Then, the FRET-efficiency is calculated by relating *I*_corr_ to the FRET-emission *I*_*F*_ and the residual portion of donor fluorescence that has not been transferred to the acceptor by FRET:

(18) $$E=\frac{I_{F}-\beta I_{D}-\alpha I_{A}}{I_{F}-\alpha I_{A}+(G-\beta )I_{D}}$$

Alternatively, *G* can be calculated based on two reference constructs of different linker length. Chen et al. (2006) applied Cerulean-Venus fusions with a linker of five (Index 1, Equation 19) and 236 amino acids (index 2, Equation 19), respectively. The acceptor emission *I*_*A*_ that is not affected by FRET was used to normalize *I*_*D*_ and *I*_corr_ for the calculation of *G* (Equation 19). Finally, the FRET-efficiency is calculated by Equation 18.

(19) $$G=\frac{\frac{I_{F1}-\alpha I_{A1}-\beta I_{D1}}{I_{A1}}-\frac{I_{F2}-\alpha I_{A2}-\beta I_{D2}}{I_{A2}}}{\frac{I_{D2}}{I_{A2}}-\frac{I_{D1}}{I_{A1}}}$$

In the other case, the FRET-efficiency is given by the ratio of the donor emission *I*_*D*_ in the presence of the acceptor and the emission of the donor in the absence of the acceptor (Hoppe et al., 2002):

(20) $$E=1-\frac{I_{D}}{\left(I_{F}-\beta I_{D}-\alpha I_{A}\right)\xi +I_{D}}$$

Based on a reference construct, ξ is determined by back calculation. In this case the determinant *E* is known as well as the correction factors α and β so that ξ is the only remaining unknown factor, if FRET-measurements are performed with the reference construct expressed in plant cells to obtain *I*_*D*_, *I*_*F*_, and *I*_*A*_. Thus, ξ is given by Equation 21:

(21) $$\xi =\frac{I_{D}E}{(1-E)\left(I_{F}-\alpha I_{A}-\beta I_{D}\right)}$$

Since *G* is used for calculating the sensitized emission based on the donor fluorescence and ξ serves for the calculation of quenched donor fluorescence based on sensitized emission, their relationship is given by:

(22) $$G=\xi^{-1}$$

Hoppe et al. (2002) further suggested a comprehensive approach to calculate the donor and acceptor fractions in the complex and the ratio of both fluorophores to overcome the imperfection of two functions contributing to the apparent FRET-efficiency that are the rate of energy transfer and the fraction of complex bound donor. It should me mentioned that the initially published equations for the determination of *R*_*M*_ and *E*_*D*_ were corrected later (Beemiller et al., 2006). Basically, the ratio of donor and acceptor can be estimated. *I*_*D*_ is linearly related to the donor concentration, if the loss of energy due to FRET is considered. Applying ξ allows for the calculation of the acceptor to donor ratio *R*_*M*_:

(23) $$R_{M}=\left(\frac{\xi }{\gamma }\right)\frac{\alpha I_{A}}{\left(I_{F}-\alpha I_{A}-\beta I_{D}\right)\xi +I_{D}}$$

The ratio of ξ and γ can be replaced by the factor *k* that is estimated based on a dataset obtained with a reference construct of known stoichiometry and calculated by Equation 24 (Chen et al., 2006).

(24) $$k=\frac{\gamma }{\xi }=\;\frac{I_{D}+\frac{\left(I_{F}-\alpha I_{A}-\beta I_{D}\right)}{G}}{I_{A}}$$

Once the FRET-efficiency *E*_*c*_ of a given complex is known, the fraction of donor in complex is given by Equation 25 (modified from Hoppe et al., 2002). However, the prerequisite for the true FRET-efficiency *E*_*c*_ of the analyzed protein pair limits the application of the equation widely.

(25) $$f_{D}=\left(1-\frac{I_{D}}{\left(I_{F}-\alpha I_{A}-\beta I_{D}\right)\xi +I_{D}}\right)E_{c}^{-1}$$

The sensitized emission and FRET-efficiency are linearly related, if the sensitized emission is expressed as ratio of acceptor emission in the presence and absence of the donor. In this case, the proportionality factor is given by the ratio of the absorption coefficients of acceptor and donor at donor's excitation wavelength (Gadella et al., 1999).

(26) $$E=\frac{\varepsilon_{A}}{\varepsilon_{D}}\left(\frac{I_{F}-\beta I_{D}}{\alpha I_{A}}-1\right)=\;\gamma \left(\frac{I_{F}-\beta I_{D}}{\alpha I_{A}}-1\right)$$

This ratio is principally known and relates the energy absorbed by the donor and transferred to the acceptor to the energy that is directly absorbed by the acceptor (Hoppe et al., 2002). For mTurquoise2 and Venus the absorption coefficients at 458 nm are 28,400 Mol^−1^ cm^−1^ and 16,300 Mol^−1^ cm^−1^, so that the ratio is 0.574. For ECFP and EYFP the absorption coefficients at 458 nm are 30,700 and 14,700 Mol^−1^ cm^−1^, respectively, resulting in the ratio 0.479 that is close to an experimentally determined γ of 0.47. However, the theoretical ratio can differ from the real situation in the cell due to the cellular and subcellular environment and its influence on the absorption.

The advantage of this method is its low dependency on differences of filter sets since mainly the emission of the acceptor is considered. Problematic is the requirement for direct excitation of the acceptor as described in the context of Equation 11 that relies on Equation 26.

If the ratio of the absorption coefficients is expressed by the single factor γ, this can be estimated based on a reference construct similar to the calculation of ξ and γ is given by Equation 27:

(27) $$\gamma =\frac{\varepsilon_{A}}{\varepsilon_{D}}=\frac{E}{\left(\frac{I_{F}-\beta I_{D}}{\alpha I_{A}}\right)-1}$$

**Figure 15:**
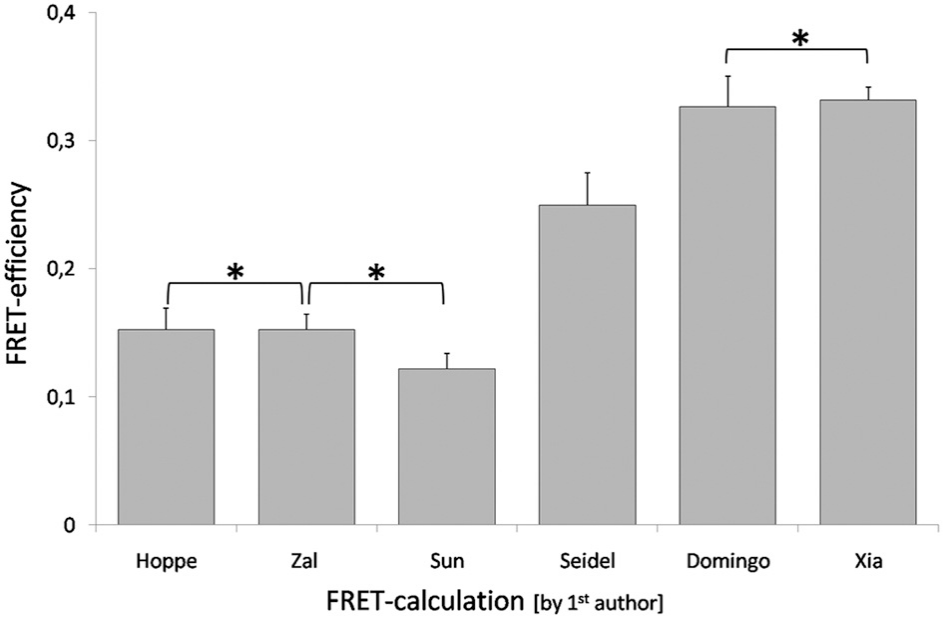
**Differences in calculation of FRET-efficiency.** The FRET-efficiency was calculated applying data recently published for the interaction between p50 and RelA in plant cells (Wolf et al., 2013b). The FRET-efficiency was calculated by six different methods, the labels correspond to the first authors of the publications that suggested the corresponding equations. An asterisk marks FRET-efficiencies that were not significantly different. The data was analyzed by student's *t*-test. The dataset comprised 22 measurements and mean ± SE are given.

The recently published data set on the dimer formation of the human transcription factors p50 and RelA in plant cells (Wolf et al., 2013b) was used for calculation of FRET-efficiencies applying the Equation 10 (Domingo et al., 2007), Equation 12 (Seidel et al., 2005), Equation 13 (Xia and Liu, 2001), Equation 18 (Zal and Gascoigne, 2004), Equation 20 (Hoppe et al., 2002), and Equation 16 (Sun and Periasamy, 2010). The non-calibrated apparent FRET-efficiencies were higher than the calibrated. The Equations 10 and 13 resulted in FRET-efficiencies of 0.33, Equation 12 in a lower apparent FRET-efficiency of 0.25. The calibrated FRET-efficiencies were in the same range and statistical analysis did not reveal significant differences among the results. The Equations 18 and 20 gave an identical FRET-efficiency of 0.15, whereas the calculation by Equation 16 gave 0.12 (Figure 15). The values for *G* and ξ were calculated applying a reference construct and were found to be 1.89 and 0.53, respectively. The variability of non-calibrated values and the congruency of the calibrated values indicate the reliability and applicability of the calibrated equations. The acceptor based equation 26 failed due to a low α-value of 0.084 that lead to a FRET-efficiency > 1.

For completeness, instead of calculation with the average emission intensities of ROIs or line profiles Heinze et al. (2013) recruited the images directly for quantification and performed pixel-wise calculation of the intensity values of whole images: First of all they divided the FRET image by the donor image and multiplied the result with 100. Next, the obtained image was divided by the donor image and multiplied with 100 again. Finally, the resulting image is divided by the acceptor image, multiplied with 30 and the result is given as intensity-encoded FRET-image. The advantage of this method is the visualization of FRET with respect to the subcellular localization of the analyzed interaction.

Quantification tools of the control software for confocal laser scanning microscopes usually provide text files. The data is either given by statistics of pixel values in a defined region or as an intensity profile along a line through the image. For analysis in plant cell, the usage of enlarged regions of interest is hampered by the central vacuole occupying at least 80 percent of the cellular volume in mature cells. In this case line intensity profiles are of advantage, if spatial correlation of the peaks in the individual channels is considered. To this end a PERL-script has been developed that calculates the FRET-efficiency based on four data sets: (i) dataset from cells expressing the donor to obtain β, (ii) dataset from cells expressing the acceptor to obtain α, (iii) dataset from cells expressing the reference constructs that is required to calculate γ, ξ or G, and finally (iv) the dataset that comprises the measurement of interest. For each dataset a folder has to be created that is named “donor,” “acceptor,” “reference,” or “measurement,” respectively. Each folder contains line profiles characterized by only one maximum. The identified maxima in the individual channels are checked for spatial correlation and background noise caused by increased “offset”-values is considered by the intensity of the first data point of each line. For the determination of the correction factors the median was applied instead of the arithmetic mean to rule out overrepresentation of outliers. The output of the PERL-script comprises the median of FRET-efficiencies that are calculated by applying equations 10, 12, 13, 18, 20, and 26. The *R*_*M*_-value is calculated by Equation 23.

## Conclusions and future perspectives

Fluorescent proteins allowed for the analysis of protein interactions in living plant cells, either by bimolecular fluorescence complementation or by FRET (Bhat et al., 2006). In the recent years, much effort has been invested in the improvement of these methods and their applicability was vastly improved, although multiple protocols and equations were published in particular for FRET that turned the situation confusing for researchers that want to start with FRET-experiments. Measuring FRET by sensitized emission has the advantage of minimal requirements for the equipment and the possibility to monitor fast intracellular processes. The drawback is its strong dependency on the characteristics of filter sets and detectors. Depending on the aim of the study, it might be sufficient to calculate apparent FRET-efficiency e.g., for documentation of conformational alterations, whereas the comparison of datasets obtained with distinct instruments demands calibration. The obtained FRET-efficiencies further depend on the photostability and spectral properties of the fluorescent protein FRET-pair, so that discussing distances instead of FRET-efficiencies compensates for different FRET-pairs and their characteristic *R*_0_-value.

The complex-formation by proteins is not limited to the interaction of two proteins, but often involves multiple proteins. One current task is the establishment of methods that allow for real time *in vivo* analysis of such complexes. The combination of BiFC and FRET is promising and has been successfully applied to detect ternary complexes of SNAREs by BiFC and FRET between cerulean as donor and folded split-YFP as acceptor (Kwaaitaal et al., 2010). Since BiFC is not limited to proteins of the avGFP-family but also has been demonstrated for mLumin, mRFP1, and mCherry (Jach et al., 2006; Fan et al., 2007; Chu et al., 2009), the combination of BiFC and FRET might be able for both donor and acceptor, if the ground lying requirement is matched that members of the avGFP family are not capable to complement with fragments of other fluorescent proteins, even if the product is not fluorescent. Otherwise, the results would become difficult to interpret.

On the other hand, an advantage of FRET is the ability to supplement a given FRET-pair by additional fluorophores toward a stepwise cascade of energy transfer between three and more fluorophores. This allows for the detection of complex formation by more than two proteins. FRET-cascades are also suitable for long range interactions (Haustein et al., 2003). FRET between three fluorescent proteins has been described before and was denominated 2-step FRET or 3-chromophore FRET (Watrob et al., 2003; Galperin et al., 2004; He et al., 2004, 2005; Seidel et al., 2010; Sun et al., 2010). For such 2-step FRET-measurements in living cells the commonly used FRET-pair CFP-YFP was supplemented by the red fluorescent proteins HcRed (He et al., 2004), mRFP1 (Galperin et al., 2004; He et al., 2005) or mCherry (Seidel et al., 2010). So the energy is ideally transferred from the donor (CFP) via the mediator (YFP) to the acceptor (red fluorescent protein), both steps were analyzed by sensitized emission. However, the situation becomes complex since three distinct FRET-pairs have to be considered, resulting e.g., in direct energy transfer between donor and acceptor bypassing the mediator (Seidel et al., 2010). This can be overcome by control measurements e.g., with REACh as a “mediator” that is no longer capable to transfer energy to the acceptor (Seidel et al., 2010) or by photoswitchable mediators that allow the direct estimation of direct energy transfer between donor and acceptor, although the absence of the mediator's absorption is expected to enhance the probability of FRET between donor and acceptor. Theoretically, FRET measurements by sensitized emission have the potential to be supplied by further fluorescent proteins like UV-fluorescent proteins such as Sirius (Tomosugi et al., 2009) as donors and infrared-fluorescent proteins as acceptors resulting in a cascade of 3–4 steps of FRET.

Recently Sun et al. (2010) suggested a FLIM-FRET approach for the detection of ternary complexes. They used the fluorophores mVenus and tdTomato as spectral different acceptors for the donor mTFP that showed the lowest fluorescence lifetime in the presence of both acceptors hence reflecting both routes of relaxation. Although this approach is less suitable for the analysis of an elongated linear arrangement of fluorophores that exceed the dimensional limitation of 1.5 *R*_0_ it is suitable for the analysis of compact ternary protein complexes. However, for enlarged complexes, the second step of energy transfer decreases the fluorescent lifetime of the mediator and might prevent its saturation by the donor. This effect has to be reflected by some decrease in the fluorescent lifetime of the donor as well. Since energy that is transferred from mVenus to tdTomato is hard to assign to the decrease in mTFP fluorescence lifetime, Sun et al. (2010) additionally applied spectral imaging and linearly unmixing for the approximation of 2-step FRET. Nevertheless, for the proof, if complexes are formed by more than two proteins, the recruitment of identical fluorescent proteins as additional acceptors should be sufficient for an analysis by FLIM as indicated by donor's lifetimes of the reference constructs consisting of two copies of Venus and one Cerulean (Chen et al., 2007; Koushik et al., 2009).

## Materials and methods

### Isolation and transfection of protoplasts

*A. thaliana* (Columbia) was grown in soil-culture in a growth chamber with 12 h light (240 μmol quanta m^−2^ s^−1^, 19°C) and 12 h dark (18°C) with 60% relative humidity. For protoplast isolation *A. thaliana* leaves were harvested from soil grown plants at the age of about 4 weeks. The isolation and the polyethylene glycol mediated transfection of *A. thaliana* protoplasts were performed as described before (Seidel et al., 2004).

### Heterologous expression of fluorescent proteins

ECFP, mTurquoise2, EYFP, mVenus, mCherry, Dronpa as well as the fusion proteins ECFP-EYFP, mTurquoise2-mVenus, Dronpa-mCherry and EYFP-mCherry were heterologously expressed in *E. coli* strain BL21 pLys (DE3). The cultures were grown to OD_600_ = 0.6 and expression was induced by supplementing the medium with 1 mM IPTG. Expression was carried out at room temperature overnight. Cells were lysed by lysozyme, treated with supersonic and cell debris were removed by centrifugation at 10,000 g for 30 min. The oligohistidine-tagged proteins were purified by Ni-NTA-affinity chromatography and finally dialyzed against 40 mM phosphate buffer pH 7.

### Spectral analysis of recombinant fluorescent proteins

FRET-efficiencies were determined via the reduced fluorescence emission of the donor. The fluorescence and absorption spectra were taken at room temperature (23°C) with a Kontron SFM25 fluorescence spectrometer and a Shimadzu UV-2401 UV/VIS spectrometer, respectively. All measurements were performed in 40 mM potassium phosphate at pH 7. For comparison of CFPs and cyan-yellow fluorescent fusion proteins the molar quantities of the CFP were adjusted equally by the absorption at 400 nm.

### Calculation of R_0_

The Förster radii were calculated for the FRET-pair Dronpa/mCherry based on the recorded absorption and emission spectra. Calculation was performed as reported before (Patterson et al., 2000; Akrap et al., 2010).

### FRET measurements

For FRET measurements, a Leica TCS SP2 confocal system with 40-fold magnification (water immersion objective HCX APO L 40×/0.8W UVI, NA = 0.8) was used. The scan speed was 400 Hz, the image resolution 1024 × 1024 pixels and 12 bit scanning mode was chosen to improve the signal to noise ratio. The transfer efficiency between the fluorophores ECFP, EYFP, mTurquoise2, mVenus, Dronpa, and mCherry was measured within mesophyll protoplasts by sensitized acceptor emission. Emission intensities were recorded sequentially (line by line) in three channels involving photomultipliers 2 and 3 and the excitation wavelengths 458 and 514 nm for cyan and YFP couples as described before (Seidel et al., 2005). PMT 2 detected the ECFP emission in the range of 470–510 nm, PMT 3 the EYFP emission in the range of 530–600 nm. For FRET-couples with mCherry the 488 nm laser line was used for excitation of Dronpa and the 543 nm laser line for excitation of mCherry. Dronpa was detected in the range of 500–530 nm, mCherry between 570 and 620 nm.

### Conflict of interest statement

The authors declare that the research was conducted in the absence of any commercial or financial relationships that could be construed as a potential conflict of interest.

## References

[B1] Agati G. (1998). Response of the *in vivo* chlorophyll fluorescence spectrum to environmental factors and laser excitation wavelength. Pure Appl. Opt. 7, 797–807 10.1088/0963-9659/7/4/016

[B2] Ai H. W. Henderson J. N. Remington S. J. Campbell R. E. (2006). Directed evolution of a monomeric, bright and photostable version of Clavularia cyan fluorescent protein: structural characterization and applications in fluorescence imaging. Biochem. J. 400, 531–540 10.1042/BJ20060874 16859491 PMC1698604

[B3] Akrap N. Seidel T. Barisas B. G. (2010). Förster distances for fluorescent resonance energy transfer between mCherry and other visible fluorescent proteins. Anal. Biochem. 402, 105–106 10.1016/j.ab.2010.03.026 20347671 PMC2885848

[B4] Albinsson B. Li S. M. Lundquist K. Stomberg R. (1999). The origin of lignin fluorescence. J. Mol. Struc. 508, 19–27 10.1016/S0022-2860(98)00913-2

[B5] Baird G. S. Zacharias D. A. Tsien R. Y. (2000). Biochemistry, mutagenesis, and oligomerization of DsRed, a red fluorescent protein from coral. Proc. Natl. Acad. Sci. U.S.A. 97, 11984–11989 10.1073/pnas.97.22.11984 11050229 PMC17281

[B6] Bayle V. Nussaume L. Bhat R. A. (2008). Combination of novel green fluorescent protein mutant TSapphire and DsRed variant mOrange to set up a versatile in planta FRET-FLIM Assay. Plant Physiol. 148, 51–60 10.1104/pp.108.117358 18621983 PMC2528103

[B7] Beemiller P. Hoppe A. D. Swanson J. A. (2006). A phosphatidylinositol-3-kinase-dependent signal transition regulates ARF1 and ARF6 during FCγ receptor-mediated phagocytosis. PLoS Biol. 4:e162 10.1371/journal.pbio.0040162 16669702 PMC1457017

[B8] Bhat R. A. (2009). FRET and FLIM applications in plants. Lab. Tech. Biochem. Mol. Biol. 33, 413–446 10.1016/S0075-7535(08)00010-7

[B9] Bhat R. A. Lahaye T. Panstruga R. (2006). The visible touch: in planta visualization of protein-protein interactions by fluorophore-based methods. Plant Methods 2:12 10.1186/1746-4811-2-12 16800872 PMC1523328

[B10] Blum C. Mexiner A. J. Subramaniam V. (2011). Dark proteins disturb multichromophore coupling in tetratmeric fluorescent proteins. J. Biophotonics 4, 114–121 10.1002/jbio.201000075 20635430

[B11] Campbell R. E. Tour O. Palmer A. E. Steinbach P. A. Baird G. S. Zacharias D. A. (2002). A monomeric red fluorescent protein. Proc. Natl. Acad. Sci. U.S.A. 99, 7877–7882 10.1073/pnas.082243699 12060735 PMC122988

[B12] Chaudhuri B. Hörmann F. Frommer W. B. (2011). Dynamic imaging of glucose flux impedance using FRET-sensors in wild-type Arabidopsis plants. J. Exp. Bot. 62, 2411–2417 10.1093/jxb/erq444 21266495

[B13] Chen Y. E. Mauldin J. P. Day R. N. Periasamy A. (2007). Characterization of spectral FRET imaging microscopy for monitoring nuclear protein interactions. J. Microsc. 228, 139–152 10.1111/j.1365-2818.2007.01838.x 17970914 PMC2874973

[B14] Chen H. Puhl H. L. Koushik S. V. Vogel S. S. Ikeda S. R. (2006). Measurement of FRET efficiency and ratio of donor to acceptor concentration in living cells. Biophys. J. 91, L39–L41 10.1529/biophysj.106.088773 16815904 PMC1544280

[B15] Cheung H. C. (1991). Resonance energy transfer, in Topics in Fluorescence Spectroscopy, Vol. 2, ed Lakowicz J. R. (New York, NY: Plenum Press), 128–176

[B16] Chu J. Zhang Z. Zheng Y. Yang J. Qin L. Lu J. (2009). A novel far-red bimolecular fluorescence complementation system that allows for efficient visualization of protein interactions under physiological conditions. Biosens. Bioelectron. 25, 234–239 10.1016/j.bios.2009.06.008 19596565

[B17] Clayton A. H. Klonis N. Cody S. H. Nice E. C. (2005). Dual-channel photobleaching FRET-microscopy for improved resolution of protein association states in living cells. Eur. Biophys. J. 34, 82–90 10.1007/s00249-004-0427-y 15232659

[B18] Clegg R. M. (2009). Förster resonance energy transfer- FRET what it is, why do it, and how it's done. Lab. Tech. Biochem. Mol. Biol. 33, 1–58 10.1016/S0075-7535(08)00001-6

[B19] Crosby K. C. Petraczewska-Bogiel A. Gadella T. W. J. Winkel B. S. (2011). Förster resonance energy transfer demonstrates a flavonoid metabolon in living plant cells that displays competitive interactions between enzymes. FEBS Lett. 585, 2193–2198 10.1016/j.febslet.2011.05.066 21669202

[B20] Cubitt A. B. Heim R. Adams S. R. Boyd A. E. Gross L. A. Tsien R. Y. (1995). Understanding, improving and using green fluorescent proteins. Trends Biochem. Sci. 20, 448–455 10.1016/S0968-0004(00)89099-4 8578587

[B21] Daelemans D. Costes S. V. Cho E. H. Erwin-Cohen R. A. Locket S. Pavlakis G. N. (2004). *In vivo* HIV-1 Rev multimerization in the nucleolus and cytoplasm identified by fluorescence resonance energy transfer. J. Biol. Chem. 279, 50167–50175 10.1074/jbc.M407713200 15294891

[B22] Dinant C. van Royen M. E. Vermeulen W. Houtsmuller A. B. (2008). Fluorescence resonance energy transfer of GFP and YFP by spectral imaging and quantitative acceptor bleaching. J. Microsc. 231, 97–104 10.1111/j.1365-2818.2008.02020.x 18638193

[B23] Dixit R. Cyr R. (2003). Cell damage and reactive oxygen species production induced by fluorescence microscopy: effect on mitosis and guidelines for non-invasive fluorescence microscopy. Plant J. 36, 280–290 10.1046/j.1365-313X.2003.01868.x 14535891

[B24] Djikanović D. Kalauzi A. Jeremić M. Mićić M. Radotić K. (2007). Deconvolution of fluorescence spectra: contribution to the structural analysis of complex molecules. Colloids Surf. B Biointerfaces 54, 188–192 10.1016/j.colsurfb.2006.10.015 17134884

[B25] Domingo B. Sabariegos R. Picazo F. Llopis J. (2007). Imaging FRET standards by steady-state fluorescence and lifetime methods. Microsc. Res. Tech. 70, 1010–1021 10.1002/jemt.20509 17722057

[B26] Duncan R. R. (2006). Fluorescence lifetime imaging microscopy (FLIM) to quantify protein-protein interactions inside cells. Biochem. Soc. Transactiv. 34, 679–682 10.1042/BST0340679 17052173 PMC1855982

[B27] Eilert U. Wolters B. Constabel F. (1986). Ultrastructure of acridine alkaloid idioblasts in roots and cell cultures of *Ruta graveolens*. Can. J. Bot. 64, 1089–1096 10.1139/b86-149

[B28] Erickson M. G. Moon D. L. Yue D. T. (2003). DsRed as a potential FRET partner with CFP and YFP. Biophys. J. 85, 599–611 10.1016/S0006-3495(03)74504-4 12829514 PMC1303115

[B29] Fan J. Y. Cui Z. Q. Wei H. P. Zhang Z. P. Zhou Y. F. Wang Y. P. (2007). Split mCherry as a new red bimolecular fluorescence complementation system for visualising protein-protein interactions in living cells. Biochem. Biophys. Res. Commun. 367, 47–53 10.1016/j.bbrc.2007.12.101 18158915

[B30] Förster T. (1946). Energiewanderung und Fluoreszenz. Naturwiss 33, 166–175 10.1007/BF00585226

[B31] Förster T. (1948). Intermolecular energy migration and fluorescence. Ann. Phys. 2, 55–75 10.1002/andp.19484370105

[B32] Fredj A. Pasquier H. Demachy I. Jonasson G. Levy B. Derrien V. (2012). The single T65S mutation generates brighter cyan fluorescent proteins with increased photostability and pH insensitivity. PLoS ONE 7:e49149 10.1371/journal.pone.0049149 23133673 PMC3487735

[B33] Gadella T. W. J. van der Krogt G. N. Bisseling T. (1999). GFP-based FRET microscopy in living plant cells. Trends Plant Sci. 4, 287–291 10.1016/S1360-1385(99)01426-0 10407445

[B34] Galperin E. Verkhusha V. V. Sorkin A. (2004). Three-chromophore FRET microscopy to analyze multiprotein interactions in living cells. Nat. Methods 1, 209–217 10.1038/nmeth720 15782196

[B35] Gandía J. Lluís C. Ferré S. Franco R. Ciruela F. (2008). Light resonance energy transfer-based methods in the study of G protein-coupled receptor oligomerization. Bioessays 30, 82–89 10.1002/bies.20682 18081019

[B36] Gandía-Herrero F. Escribano J. García-Carmona F. (2005). Betaxanthines as pigments responsible for visible fluorescence in flowers. Planta 222, 586–593 10.1007/s00425-005-0004-3 16177911

[B37] Ganesan S. Ameer-beg S. M. Ng T. T. Vojnovic B. Wouters F. S. (2006). A dark yellow fluorescent protein (YFP)-based resonance energy accepting chromoprotein (REACh) for Förster resonance energy transfer with GFP. Proc. Natl. Acad. Sci. U.S.A. 103, 4089–4094 10.1073/pnas.0509922103 16537489 PMC1449651

[B38] Gerritsen H. C. Agronskaia A. V. Bader A. N. Esposito A. (2009). Time domain FLIM: theory, instrumentation, and data analysis, in Laboratory Techniques in Biochemistry and Molecular Biology, Vol. 33, ed Gadella T. W. J. (Oxford: Elsevier), 95–132

[B39] Gillbro T. Cogdell R. J. (1989). Carotenoid fluorescence. Chem. Phys. Lett. 158, 312–316 10.1016/0009-2614(89)87342-7

[B40] Goedhart J. van Weeren L. Hink M. A. Vischer N. O. Jalink K. Gadella T. W. J. (2010). Bright cyan fluorescent protein variants ideintified by fluorescence lifetime screening. Nat. Methods 7, 137–139 10.1038/nmeth.1415 20081836

[B41] Goedhart J. Vermeer J. E. M. Adjobo-Hermans M. J. W. van Weeren L. Gadella T. W. J. (2007). Sensitive detection of p65 homodimers using red-shifted and fluorescent protein-based FRET-couples. PLoS ONE 10:e1011 10.1371/journal.pone.0001011 17925859 PMC1995760

[B42] Goedhart J. von Stetten D. Noirclerc-Savoye M. Lelimousin M. Joosen L. Hink M. A. (2012). Structure-guided evolution of cyan fluorescent proteins towards a quantum yield of 93%. Nat. Commun. 3, 751 10.1038/ncomms1738 22434194 PMC3316892

[B43] Gordon G. W. Berry G. Liang X. H. Levine B. Herman B. (1998). Quantitative fluorescence resonance energy transfer measurements using fluorescence microscopy. Biophys. J. 74, 2702–2713 10.1016/S0006-3495(98)77976-7 9591694 PMC1299610

[B44] Griesbeck O. Baird G. S. Campbell R. E. Zacharias D. A. Tsien R. Y. (2001). Reducing the environmental sensitivity of yellow fluorescent protein. Mechanism and application. J. Bio. Chem. 276, 29188–29194 10.1074/jbc.M102815200 11387331

[B45] Guex N. Peitsch M. C. (1997). SWISS-MODEL and the Swiss-Pdb-Viewer: an environment for comparative protein modeling. Electrophoresis 18, 2714–2723 10.1002/elps.1150181505 9504803

[B46] Haseloff J. Siemering K. R. Prasher D. C. Hodge S. (1997). Removal of a cryptic intron and subcellular localization of green fluorescent protein are required to mark transgenic Arabidopsis plants brightly. Proc. Natl. Acad. Sci. U.S.A. 94, 2122–2127 10.1073/pnas.94.6.2122 9122158 PMC20051

[B47] Haustein E. Jahnz M. Schwille P. (2003). Triple FRET: A tool for studying long-range molecular interactions. Chem. Phys. Chem. 4, 745–748 10.1002/cphc.200200634 12901306

[B48] He L. Grammer A. C. Wu X. Lipsky P. E. (2004). TRAF3 forms heterotrimers with TRAF2 and modulates its ability to mediate NF-(kappa)B activation. J. Biol. Chem. 279, 55855–55865 10.1074/jbc.M407284200 15383523

[B49] He L. Wu X. Simone J. Hewgill D. Lipsky P. E. (2005). Determination of tumor necrosis factor receptor-associated factor trimerization in living cells by CFP->YFP->mRFP FRET detected by flow cytometry. Nucleic Acid Res. 33, e61 10.1093/nar/gni057 15805120 PMC1074310

[B50] Heikal A. A. Hess S. T. Baird G. S. Tsien R. Y. Webb W. W. (2000). Molecular spectroscopy and dynamics of intrinsically fluorescent proteins: coral red (dsRed) and yellow (Citrine). Proc. Natl. Acad. Sci. U.S.A. 97, 11996–12001 10.1073/pnas.97.22.11996 11050231 PMC17283

[B51] Heinze M. Herre M. Massalski C. Hermann I. Conrad U. Roos W. (2013). Signal transfer in the plant plasma membrane: phospholipase A2 is regulated via an inhibitory Gα protein and a cyclophylin. Biochem. J. 450, 497–509 10.1042/BJ20120793 23252374

[B52] Hink M. A. Bisseling T. Visser A. J. (2002). Imaging protein-protein interactions in living plant cells. Plant Mol. Biol. 50, 871–883 10.1023/A:1021282619035 12516859

[B53] Hoppe A. D. Christensen K. Swanson J. A. (2002). Fluorescence Resonance Energy Transfer-based stoichiometry in living cells. Biophys. J. 83, 3652–3664 10.1016/S0006-3495(02)75365-4 12496132 PMC1302440

[B54] Hossain T. Rosenberg I. Selhub J. Kishore G. Beachy R. Schubert K. (2004). Enhancement of folates in plants through metabolic engineering. Proc. Natl. Acad. Sci. U.S.A. 101, 5158–5163 10.1073/pnas.0401342101 15044686 PMC387390

[B55] Jach G. Pesch M. Richter K. Frins S. Uhrig J. F. (2006). An improved mRFP1 adds red to bimolecular fluorescence complementation. Nat. Methods 3, 597–600 10.1038/nmeth901 16862132

[B56] Jalink K. van Rheenen J. (2009). Filter FRET: quantitative imaging of sensitized emission, in Laboratory Techniques in Biochemistry and Molecular Biology, Vol. 33, ed Gadella T. W. J. (Oxford: Elsevier), 289–350

[B57] Jares-Erijman E. A. Jovin T. M. (2003). FRET imaging. Nat. Biotechnol. 21, 1387–1395 10.1038/nbt896 14595367

[B58] Karpova T. S. Baumann C. T. He L. Wu X. Grammer A. Lipsky P. (2003). Fluorescence resonance energy transfer from cyan to yellow fluorescent protein detected by acceptor photo-bleaching using confocal microscopy and a single laser. J. Microsc. 209, 56–70 10.1046/j.1365-2818.2003.01100.x 12535185

[B59] Klein P. Seidel T. Stöcker B. Dietz K. J. (2012). The membrane-tethered transcription factor ANAC089 serves as redox-dependent suppressor of stromal ascorbate peroxidase gene expression. Front. Plant Sci. 3:247 10.3389/fpls.2012.00247 23162559 PMC3493970

[B60] Kleinegris D. van Es M. A. Janssen M. Brandenburg W. A. Wijffels R. H. (2010). Carotenoid fluorescnece in *Dunaliella salina*. J. Appl. Phycol. 22, 645–649 10.1007/s10811-010-9505-y 20835349 PMC2935544

[B61] Kluge C. Seidel T. Bolte S. Sharma S. S. Hanitzsch M. Satiat-Jeunemaitre B. (2004). Subcellular distribution of the V-ATPase complex in plant cells, and *in vivo* localisation of the 100 kDa subunit VHA-a within the complex. BMC Cell Biol. 5:29 10.1186/1471-2121-5-29 15310389 PMC516168

[B62] Koushik S. V. Blank P. S. Vogel S. S. (2009). Anomalous surplus energy transfer observed with multiple FRET-acceptors. PLoS ONE 4:e8031 10.1371/journal.pone.0008031 19946626 PMC2778011

[B63] Koushik S. V. Chen H. Thaler C. Puhl H. L. Vogel S. S. (2006). Cerulean, Venus and VenusY67C FRET reference standards. Biophys. J. 91, L99–L101 10.1529/biophysj.106.096206 17040988 PMC1779932

[B64] Krebs M. Held K. Binder A. Hashimoto K. Den Herder G. Parniske M. (2012). FRET-based genetically encoded sensors allow high-resolution live cell imaging of Ca^2+^-dynamics. Plant J. 69, 181–192 10.1111/j.1365-313X.2011.04780.x 21910770

[B65] Kremers G. J. Goedhart J. (2009). Visible fluorescent proteins for FRET. Lab. Tech. Biochem. Mol. Biol. 33, 171–224 10.1016/S0075-7535(08)00005-3

[B66] Kremers G. J. Goedhart J. van Munster E. B. Gadella T. W. J. (2006). Cyan and yellow fluorescent proteins with improved brightness, protein folding, and FRET Förster radius. Biochem. 45, 6570–6580 10.1021/bi0516273 16716067

[B67] Kwaaitaal M. Keinath N. F. Pajonk S. Biskup C. Panstruga R. (2010). Combined bimolecular fluorescence complementation and Förster resonance energy transfer reveals ternary SNARE complex formation in living plant cells. Plant Physiol. 152, 1135–1147 10.1104/pp.109.151142 20071602 PMC2832253

[B68] Lakowicz J. R. (2006). Principles of Fluorescent Spectroscopy, 3rd Edn. New York, NY: Springer 10.1007/978-0-387-46312-4

[B69] Lam A. St-Pierre F. Gong Y. Marshall J. D. Cranfill P. J. Baird M. A. (2012). Improving FRET dynamic range with bright green and red fluorescent protein. Nat. Methods 9, 1005–1012 10.1038/nmeth.2171 22961245 PMC3461113

[B70] Lidke D. S. Nagy P. Barisas B. G. Heintzmann R. Post J. N. Lidke K. A. (2003). Imaging molecular interactions in cells by dynamic and static fluorescence anisotropy (rFLIM and emFRET). Biochem. Soc. Trans. 31, 1020–1027 10.1042/BST0311020 14505472

[B71] Malkani N. Schmid J. A. (2011). Some secrets of fluorescent proteins: distinct bleaching in various mounting fluids and photoactivation of cyan fluorescent proteins at yfp-excitation. PLoS ONE 6:e18586 10.1371/journal.pone.0018586 21490932 PMC3072413

[B72] Markwardt M. L. Kremers G. J. Kraft C. A. Ray K. Cranfill P. J. Wilson K. A. (2011). An improved cerulean fluorescent protein with enhanced brightness and reduced reversible photoswitching. PLoS ONE 6:e17896 10.1371/journal.pone.0017896 21479270 PMC3066204

[B73] Megías D. Marrero R. Martínez del Peso B. Garcia M. A. Bravo-Cordero J. J. García-Grande A. (2009). Novel lambda FRET spectral confocal microscopy imaging method. Microsc. Res. Techn. 72, 1–11 10.1002/jemt.20633 18785251

[B74] Miyawaki A. Griesbeck O. Heim R. Tsien R. Y. (1999). Dynamic and quantitative Ca^2+^-measurements using improved cameleons. Proc. Natl. Acad. Sci. U.S.A. 96, 2134–2140 10.1073/pnas.96.5.2135 10051607 PMC26749

[B75] Miyawaki A. Tsien R. Y. (2000). Monitoring protein conformations and interactions by fluorescence resonance energy transfer between mutants of green fluorescent protein. Methods Enzymol. 327, 427–500 10.1016/S0076-6879(00)27297-2 11045004

[B76] Miyawaki A. Llopis J. Heim R. McCaffery J. M. Adams J. A. Ikura M. (1997). Fluorescent indicators for Ca^2+^ based on green fluorescent proteins and calmodulin. Nature 388, 882–887 10.1038/42264 9278050

[B77] Mizuno H. Sawano A. Eli P. Hama H. Miyawaki A. (2001). Red fluorescent protein from Discosoma as a fusion tag and a partner for fluorescence resonance energy transfer. Biochemistry. 40, 2502–2510 10.1021/bi002263b 11327872

[B78] Murakoshi H. Lee S. J. Yasuda R. (2008). Highly sensitive and quantitative FRET-FLIM imaging in single dendritic spines using improved non-radiative YFP. Brain Cell Biol. 36, 31–42 10.1007/s11068-008-9024-9 18512154 PMC2673728

[B79] Muthuramalingam M. Seidel T. Laxa M. Nunes de Miranda S. M. Gärtner F. Ströher E. (2009). Multiple redox and non-redox interactions define 2-Cys peroxiredoxin as a regulatory hub in the chloroplast. Mol. Plant 2, 1273–1288 10.1093/mp/ssp089 19995730

[B80] Nagai T. Ibata K. Park E. S. Kubota M. Mikoshiba K. Miyawaki A. (2002). A variant of yellow fluorescent protein with fast and efficient maturation for cell-biological applications. Nat. Biotechnol. 20, 87–90 10.1038/nbt0102-87 11753368

[B81] Nagai T. Yamada S. Tominaga T. Ichikawa M. Miyawaki A. (2004). Expanded dynamic range of fluorescent indicators for Ca^2+^ by circularly permuted yellow fluorescent proteins. Proc. Natl. Acad. Sci. U.S.A. 101, 10554–10559 10.1073/pnas.0400417101 15247428 PMC490022

[B82] Okumoto S. Jones A. Frommer W. B. (2012). Quantitative imaging with fluorescent biosensors. Annu. Rev. Plant Biol. 63, 663–706 10.1146/annurev-arplant-042110-103745 22404462

[B83] Otani M. Shitan N. Sakai K. Martinoia E. Sato F. Yazaki K. (2005). Characterization of vacuolar transport of the endogenous alkaloid berberine in *Coptis japonica*. Plant Physiol. 138, 1939–1946 10.1104/pp.105.064352 16024684 PMC1183385

[B84] Padilla-Parra S. Audugé N. Coppey-Moisan M. Tramier M. (2008). Quantitative FRET-analysis by fast acquisitions time domain FLIM at high spatial resolution in living cells. Biophys. J. 95, 2976–2988 10.1529/biophysj.108.131276 18539634 PMC2527249

[B85] Padilla-Parra S. Audugé N. Lalucque H. Mevel J. C. Coppey-Moisan M. Tramier M. (2009). Quantitative comparison of different fluorescent protein couples for fast FRET-FLIM acquisition. Biophys. J. 97, 2368–2376 10.1016/j.bpj.2009.07.044 19843469 PMC2764072

[B86] Patterson G. H. Piston D. W. Barisas B. G. (2000). Förster distances between green fluorescent protein pairs. Anal. Biochem. 284, 438–440 10.1006/abio.2000.4708 10964438

[B87] Peter M. Ameer-Beg S. M. Hughes M. K. Y. Keppler M. D. Prag S. Marsh M. (2005). Biophys. J. 88, 1224–1237 10.1529/biophysj.104.050153 15531633 PMC1305125

[B88] Philipps B. Hennecke J. Glockshuber R. (2003). FRET-based *in vivo* screening for protein folding and increased protein stability. J. Mol. Biol. 327, 239–249 10.1016/S0022-2836(03)00077-9 12614622

[B89] Piston D. W. Kremer G. J. (2007). Fluorescent protein FRET: the good, the bad and the ugly. Trends Biochem. Sci. 32, 407–414 10.1016/j.tibs.2007.08.003 17764955

[B90] Pöhlker C. Huffman J. A. Pöschl U. (2011). Autofluorescence of atmospheric bioaerosols – fluorescent biomolecules and potential interferences. Atmos. Meas. Tech. 4, 5857–5933 10.5194/amtd-4-5857-2011

[B89a] Poot M. Pierce R. H. Kavanagh T. J. (2002). Flow cytometric and fluorometric methods of quantifying and characterizing apoptotic cell death, in Apoptosis methods in Pharmakology and Toxikology, ed M. A. Davis (Totowa, NJ: Humana Press), 11–36

[B91] Raarup M. K. Fjorback A. W. Jensen S. M. R. Müller H. K. Kjærgaard M. M. Poulsen H. (2009). Enhanced yellow fluorescent protein photoconversion to a cyan fluorescent protein-like species is sensitive to thermal and diffusion conditions. J. Biomed. Opt. 14, 034039 10.1117/1.3103338 19566331

[B92] Raicu V. Jansma D. B. Miller R. J. D. Friesen J. D. (2005). Protein interaction quantified *in vivo* by spectrally resolved fluorescence resonance energy transfer. Biochem. J. 385, 265–277 10.1042/BJ20040226 15352875 PMC1134695

[B93] Rizzo M. A. Springer G. Segawa K. Zipfel W. R. Piston D. W. (2006). Optimization of pairings and detection conditions for measurement of FRET between cyan and yellow fluorescent proteins. Microsc. Microanal. 12, 238–254 10.1017/S1431927606060235 17481360

[B94] Rizzo M. A. Springer G. H. Granada B. Piston D. W. (2004). An improved cyan fluorescent protein variant useful for FRET. Nat. Biotechnol. 22, 445–449 10.1038/nbt945 14990965

[B95] Roshchina V. V. (2012). Vital autofluorescence: application to the study of plant living cells. Int. J. Spectrosc. 2012, 1–14 10.1155/2012/124672

[B96] Roshchina V. V. Karnaukhov V. N. (1999). Changes in pollen autofluorescence induced by ozone. Biol. Plant. 42, 273–278 10.1023/A:1002120904588

[B97] Schmid J. A. Scholze P. Kudlacek O. Freissmuth M. Singer E. A. Sitte H. H. (2001). Oligomerization of the human serotonin transporter and of the rat GABA transporter 1 visualized by fluorescence resonance energy transfer microscopy in living cells. J. Biol. Chem. 276, 3805–3810 10.1074/jbc.M007357200 11071889

[B98] Schnitzer D. Seidel T. Sander T. Golldack D. Dietz K. J. (2011). The cellular energization state affects peripheral stalk stability of plant vacuolar H^+^-ATPase and impairs vacuolar acidification. Plant Cell Physiol. 52, 946–956 10.1093/pcp/pcr044 21474463

[B99] Seefeldt B. Kasper R. Seidel T. Tinnefeld P. Dietz K. J. Heilemann M. (2008). Fluorescent proteins for single-molecule fluorescence applications. J. Biophotonics 1, 74–82 10.1002/jbio.200710024 19343637

[B100] Seidel T. Golldack D. Dietz K. J. (2005). Mapping of C-termini of V-ATPase subunits by *in vivo*-FRET measurements. FEBS Lett. 579, 4374–4382 10.1016/j.febslet.2005.06.077 16061227

[B101] Seidel T. Kluge C. Hanitzsch M. Ross J. Sauer M. Dietz K. J. (2004). Colocalization and FRET-analysis of subunits c and a of the vacuolar H^+^-ATPase in living plant cells. J. Biotech. 112, 165–175 10.1016/j.jbiotec.2004.04.027 15288951

[B102] Seidel T. Seefeldt B. Sauer M. Dietz K. J. (2010). *In vivo* analysis of the 2-Cys peroxiredoxin oligomeric state by two-step FRET. J. Biotechnol. 149, 272–279 10.1016/j.jbiotec.2010.06.016 20615439

[B103] Seward H. E. Basran J. Denton R. Pfuhl M. Muskett F. W. Bagshaw C. R. (2013). Halide and proton binding kinetics of yellow fluorescent protein variants. Biochemistry 52, 2482–2491 10.1021/bi3016839 23514090

[B104] Shaner N. C. Campbell R. E. Steinbach P. A. Giepmans B. N. Palmer A. E. Tsien R. Y. (2004). Improved monomeric red, orange and yellow fluorescent proteins derived from Discosoma sp. red fluorescent protein. Nat. Biotechnol. 22, 1567–1572 10.1038/nbt1037 15558047

[B105] Sun Y. Periasamy A. (2010). Additional correction for energy transfer efficiency calculation in filter-based resonance energy transfer microscopy for more accurate results. J. Biomed. Opt. 15, 020513 10.1117/1.3407655 20459222 PMC2874045

[B106] Sun Y. Wallrabe H. Booker C. F. Day R. N. Periasamy A. (2010). Three-color spetral FRET microscopy localizes three interacting proteins in living cells. Biophys. J. 99, 1274–1283 10.1016/j.bpj.2010.06.004 20713013 PMC2920763

[B107] Szentesi G. Vereb G. Horváth G. Bodnár A. Fábián Á. Matkó J. (2005). Computer program for analysing donor photobleaching FRET image series. Cytometry A 67A, 119–128 10.1002/cyto.a.20175 16163694

[B108] Tomosugi W. Matsuda T. Tani T. Nemoto T. Kotera I. Saito K. (2009). An ultramarine fluorescent protein with increased photostability and pH insensitivity. Nat. Methods 6, 351–353 10.1038/nmeth.1317 19349978

[B109] Tramier M. Zahid M. Mevel J. C. Masse M. J. Coppey-Moisan M. (2006). Sensitivity of CFP/YFP and GFP/mCherry pairs to donor photobleaching on FRET determination by fluorescence lifetime imaging microscopy in living cells. Microsc. Res. Tech. 69, 933–939 10.1002/jemt.20370 16941642

[B110] Valentin G. Verheggen C. Piolot T. Neel H. Coppey-Moisan M. Bertrand E. (2005). Photoconversion of YFP into a CFP-like species during acceptor photobleaching FRET experiments. Nat. Methods 2, 801 10.1038/nmeth1105-801 16278647

[B111] van Munster E. B. Kremers G. J. Adjobo-Hermans M. J. W. Gadella T. W. J. (2005). Fluorescence resonance Energy transfer (FRET) measurement by gradual acceptor photobleaching. J. Microsc. 218, 253–262 10.1111/j.1365-2818.2005.01483.x 15958019

[B112] van Rheenen J. Langeslag M. Jalink K. (2004). Correcting confocal acquisition to optimize imaging of fluorescence resonance energy transfer by sensitized emission. Biophys. J. 86, 2517–2529 10.1016/S0006-3495(04)74307-6 15041688 PMC1304099

[B113] Veerabagu M. Elgass K. Kirchler T. Huppenberger P. Harter K. Chaban C. (2012). The Arabidopsis B-type response regulator 18 homodimerizes and positively regulates cytokinin responses. Plant J. 72, 721–731 10.1111/j.1365-313X.2012.05101.x 22775331

[B114] Vermeer J. E. M. van Munster E. B. Vischer N. O. Gadella T. W. J. (2004). Probing plasma membrane microdomains in cowpea protoplasts using lapidated GFP-fusion proteins and multimode FRET microscopy. J. Microsc. 214, 190–200 10.1111/j.0022-2720.2004.01318.x 15102066

[B115] Verveer P. J. Hanley Q. S. (2009). Frequency domain FLIM theory, instrumentation, and data analysis. Lab. Tech. Biochem. Mol. Biol. 33, 59–94 10.1016/S0075-7535(08)00002-8

[B116] Vitha S. Osteryoung K. W. (2011). Immunofluorescence microscopy for localization of Arabidopsis chloroplast proteins. Methods Mol. Biol. 774, 33–58 10.1007/978-1-61779-234-2_3 21822831

[B117] Vogel S. S. Nguyen T. A. van der Meer B. W. Blank P. S. (2012). The impact of heterogeneity and dark acceptor states on FRET: implications for using fluorescent protein donors and acceptors. PLoS ONE 7:e49593 10.1371/journal.pone.0049593 23152925 PMC3496711

[B118] Wallrabe H. Periasamy A. (2005). Imaging protein molecules using FRET and FLIM microscopy. Curr. Opin. Biotechnol. 16, 19–27 10.1016/j.copbio.2004.12.002 15722011

[B119] Wanke D. Hohenstatt M. L. Dynowski M. Bloss U. Hecker A. Elgass K. (2011). Alanine zipper-like coiled-coil domains are necessary for homotypic dimerization of plant GAGA-factors in the nucleus and nucleolus. PLoS ONE 6:e16070 10.1371/journal.pone.0016070 21347358 PMC3037368

[B120] Watrob H. M. Pan C. P. Barkley M. D. (2003). Two-step FRET as a structural tool. J. Am. Chem. Soc. 125, 7336–7343 10.1021/ja034564p 12797808

[B121] Wirtz M. Beard K. F. M. Lee C. P. Boltz A. Schwarzländer M. Fuchs C. (2012). Mitochondrial cysteine synthase complex regulates O-Acetylserine biosynthesis in plants. J. Biol. Chem. 287, 27941–27947 10.1074/jbc.M112.372656 22730323 PMC3431704

[B122] Wolf H. Barisas B. G. Dietz K. J. Seidel T. (2013a). Kaede for detection of protein oligomerization. Mol. Plant. 6, 1453–1462 10.1093/mp/sst039 23430050

[B123] Wolf A. Akrap N. Marg B. Galliardt H. Heiligentag M. Humpert F. (2013b). Elements of the transcriptional machinery are compatible among plants and mammals. PLoS ONE 8:e53737 10.1371/journal.pone.0053737 23326494 PMC3543382

[B124] Wolfbeis O. S. (1985). The fluorescence of organic natural products, in Molecular Luminescence Spectroscopy: Methods and Applications, ed S. G. Schulman (New York, NY: John Wiley and Sons).

[B125] Wu B. Chen Y. Müller J. D. (2009). Fluorescence fluctuation spectroscopy of mCherry in living cells. Biophys. J. 96, 2391–2404 10.1016/j.bpj.2008.12.3902 19289064 PMC2907682

[B126] Xia Z. Liu Y. (2001). Reliable and global measurement of fluorescence resonance energy transfer using fluorescence microscopes. Biophys. J. 81, 2395–2402 10.1016/S0006-3495(01)75886-9 11566809 PMC1301710

[B127] Yang F. Moss L. G. Philipps Jr. (1996). Structure of the green fluorescent protein. Nat. Biotechnol. 14, 1246–1251 10.1038/nbt1096-1246 9631087

[B128] Zal T. Gascoigne N. R. J. (2004). Photobleaching-corrected FRET efficiency imaging of live cells. Biophys. J. 86, 3923–3939 10.1529/biophysj.103.022087 15189889 PMC1304294

[B129] Zapata-Hommer O. Griesbeck O. (2003). Efficiently folding and circularly permuted variants of the Sapphire mutant of GFP. BMC Biotechnol. 3:5 10.1186/1472-6750-3-5 12769828 PMC161811

